# Rta is the principal activator of Epstein-Barr virus epithelial lytic transcription

**DOI:** 10.1371/journal.ppat.1010886

**Published:** 2022-09-29

**Authors:** Ahmed Ali, Makoto Ohashi, Alejandro Casco, Reza Djavadian, Mark Eichelberg, Shannon C. Kenney, Eric Johannsen

**Affiliations:** 1 Department of Oncology, McArdle Laboratory for Cancer Research, University of Wisconsin, Madison Wisconsin, United States of America; 2 National Center for Research, Khartoum, Sudan; 3 Department of Medicine, Division of Infectious Diseases, University of Wisconsin, Madison, Wisconsin, United States of America; University of California, Berkeley, UNITED STATES

## Abstract

The transition from latent Epstein-Barr virus (EBV) infection to lytic viral replication is mediated by the viral transcription factors Rta and Zta. Although both are required for virion production, dissecting the specific roles played by Rta and Zta is challenging because they induce each other’s expression. To circumvent this, we constructed an EBV mutant deleted for the genes encoding Rta and Zta (BRLF1 and BZLF1, respectively) in the Akata strain BACmid. This mutant, termed EBVΔRZ, was used to infect several epithelial cell lines, including telomerase-immortalized normal oral keratinocytes, a highly physiologic model of EBV epithelial cell infection. Using RNA-seq, we determined the gene expression induced by each viral transactivator. Surprisingly, Zta alone only induced expression of the lytic origin transcripts BHLF1 and LF3. In contrast, Rta activated the majority of EBV early gene transcripts. As expected, Zta and Rta were both required for expression of late gene transcripts. Zta also cooperated with Rta to enhance a subset of early gene transcripts (Rta^synergy^ transcripts) that Zta was unable to activate when expressed alone. Interestingly, Rta and Zta each cooperatively enhanced the other’s binding to EBV early gene promoters, but this effect was not restricted to promoters where synergy was observed. We demonstrate that Zta did not affect Rta^synergy^ transcript stability, but increased Rta^synergy^ gene transcription despite having no effect on their transcription when expressed alone. Our results suggest that, at least in epithelial cells, Rta is the dominant transactivator and that Zta functions primarily to support DNA replication and co-activate a subset of early promoters with Rta. This closely parallels the arrangement in KSHV where ORF50 (Rta homolog) is the principal activator of lytic transcription and K8 (Zta homolog) is required for DNA replication at oriLyt.

## Author summary

The Epstein-Barr virus (EBV) replication is controlled by the Rta and Zta viral transcription factors. Because Rta and Zta each induce the other’s expression, prior studies have largely focused on the extent to which Rta versus Zta can initiate the replication cascade in a particular cell type. These experiments, while important, are largely determined by the efficiency by which Rta induces expression of Zta and conversely, how well Zta induces Rta expression. Consequently, much of what we know about the specific roles of Rta versus Zta in EBV replication derives from reporter gene assays which are subject to several types of artifacts. Using epithelial cells infected with an EBV mutant deleted for Rta and Zta (EBVΔRZ), we were able to assess the responsiveness of all viral lytic promoters to Rta alone or Zta alone. Unexpectedly, we found that Zta played a much more restricted role than expected and that Rta was the principal activator of EBV early lytic genes. Our findings suggest that transcriptional control of EBV replication in epithelial cells more closely mirrors that of the Kaposi Sarcoma Herpesvirus (KSHV) than previously appreciated.

## Introduction

Epstein-Barr virus (EBV) is a ubiquitous human γ-herpesvirus which infects ~95% of the world’s population. EBV is associated with B-cell lymphomas and epithelial malignancies, including nasopharyngeal and gastric carcinomas [1]. Like all members of the Herpesviridae family, EBV undergoes two distinct modes of infection in cells, latent and lytic. During latency, few viral genes are expressed, and the viral genome undergoes licensed DNA replication by the host machinery once per cell cycle. EBV-associated malignancies are characterized by latent infection, although a small minority of tumor cells may express lytic gene products (reviewed in [2]).

The lytic cycle of replication culminates with production of virion particles typically in oral epithelial cells, but is also the means by which EBV emerges from its *in vivo* latency reservoir, the B lymphocyte. The lytic cascade involves expression of ~80 genes and there is increasing evidence that, in addition to being essential for viral spread, lytic gene expression contributes to the development of EBV-associated malignancies [3–5]. Multiple different stimuli have been reported to disrupt EBV latency, but cellular differentiation appears to be the most important initiator of the lytic cascade *in vivo* [6,7]. Lytic induction can be accomplished *in vitro* by phorbol esters, calcium ionophores, histone deacetylase inhibitors, DNA demethylating agents, anti-immunoglobulin, and transforming growth factor-beta. The key event that these agents must incite to disrupt latency is transcription of the master lytic transcription factors Rta and Zta, encoded by EBV genes BRLF1 and BZLF1, respectively (see [8] for review).

Zta is a basic leucine zipper (bZIP) transcription factor that binds to sites known as Zta responsive elements (ZREs) which include sequences matching consensus AP-1 sites. Zta is unique among bZIP transcription factors in that it can also bind to CpG containing ZRE sequences in a methylation dependent manner. [9–17] In addition to its role in lytic gene expression, Zta also functions as an oriLyt binding protein that is essential for viral DNA replication [18,19]. This activity is shared with its KSHV ortholog K8, but the latter, unlike Zta does not play a role in activation of lytic gene promoters [20].

The Rta transcription factor is without known metazoan homologs, but has orthologs among all gammaherpesviruses (i.e., the ORF50 transcription factors). Rta binds to multiple cognate DNA sites present in the EBV genome called Rta responsive elements (RREs) [21–23]. It can also activate promoters lacking RREs indirectly via activation of cytoplasmic signaling cascades [24,25], or possibly by indirect binding to DNA via interactions with various host proteins such as Sp1, Sp3, and MCAF1 [26,27].

Although Rta and Zta are both essential for completion of the lytic cascade [28], their ability to initiate it varies with cell type. This is due to their dependence upon cell specific cofactors and epigenetic modifiers to activate each other’s transcription. For example, Zta’s dependence upon CpG methylation makes Rta the superior lytic induction agent in NOK cells, when the EBV genome, and in particular the Rta promoter, is hypomethylated [29]. By contrast, Zta is superior at inducing the lytic cascade in many tumor cell lines when the genome is heavily methylated [29–31]. These experiments, while important, largely reflect the ability of either Rta or Zta to induce each other’s promoters and shed little light on the specific roles played by each transcription factor in the lytic cascade. Although the responsiveness of lytic promoters to Rta alone or Zta alone has been characterized in reporter assays [32–36], the degree to which these promoters are activated by Rta alone or Zta alone in the context of the viral genome is unknown for all but a few lytic genes. In order to address this gap in our knowledge, we constructed an EBV genome deleted for BRLF1 and BZLF1 in the Akata Bacmid (EBVΔRZ) and characterized the dependence of lytic transcription upon Rta and Zta in multiple cell types by RNA-seq.

## Results

### Distinct roles of Rta and Zta in activating EBV lytic gene expression

Dissecting the specific roles played by Rta and Zta during EBV lytic replication is challenging because they activate one another’s expression and cooperatively activate many lytic promoters [28,34,36]. To circumvent this, we employed an EBV mutant deleted for both the BRLF1 and BZLF1 genes in the Akata strain BACmid (designated EBVΔRZ). To characterize this mutant, we initially infected HeLa cells with EBVΔRZ utilizing BM2710 invasive E. coli and trans-complemented them with Zta and Rta. Supernatants from these trans-complemented HeLa-EBVΔRZ cells were then used to infect the EBV negative Burkitt lymphoma Akata cell line (S1 Fig), demonstrating that EBVΔRZ only requires exogenous Rta and Zta expression to restore replication competence.

**Fig 1:**
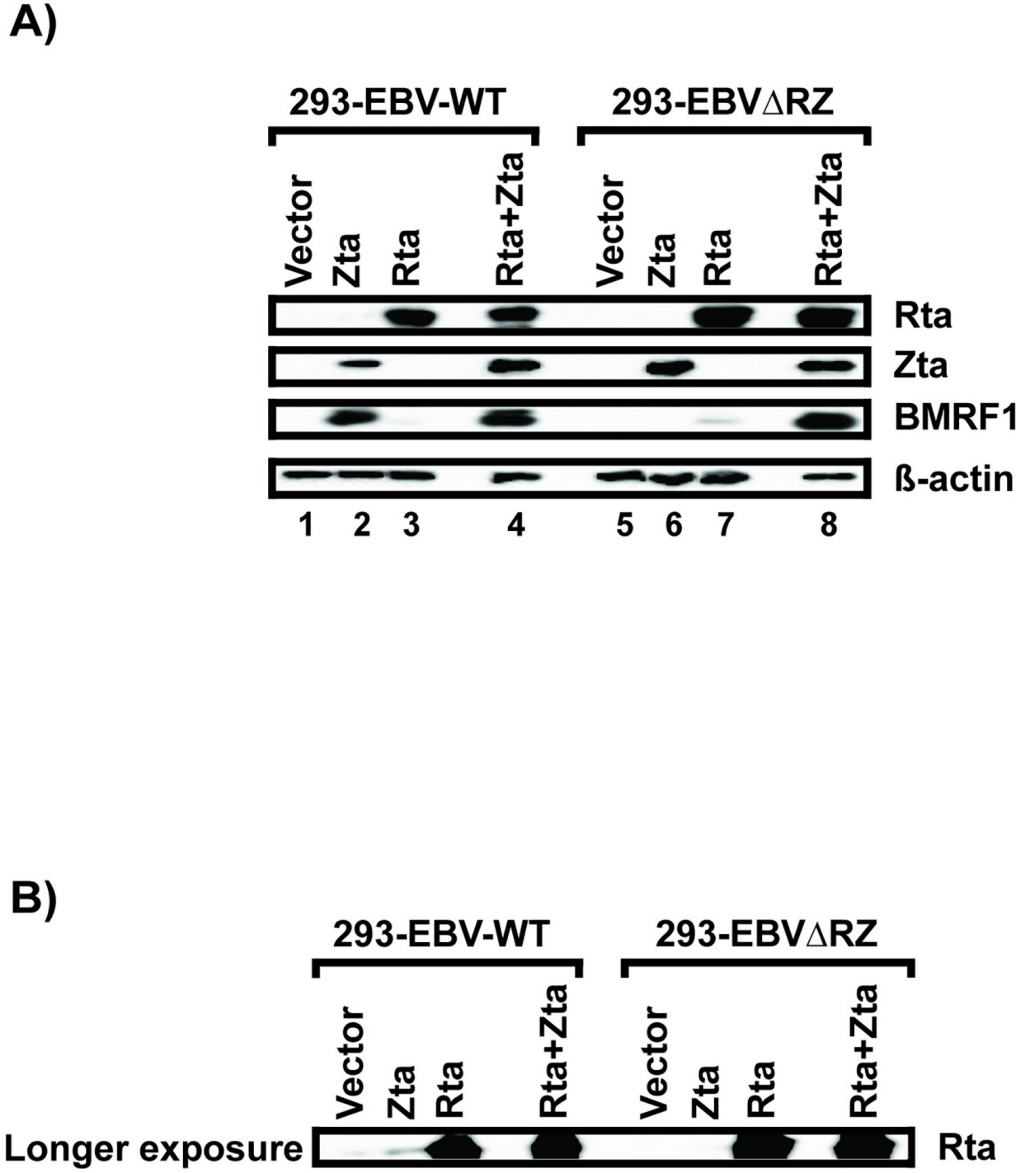
EBV deleted for IE locus allows independent assessment of the role of Rta and Zta in lytic gene regulation. (A) Western blots probed for the indicated EBV, or cell proteins of lysates prepared from 293 cells infected with WT Akata (239-EBV-WT) or AkataΔRZ virus (293-EBVΔRZ) transfected with empty vector, or expression plasmid for Zta, Rta, or expression plasmids for both Rta and Zta (Rta+Zta). Data are representative of two independent experiments. Lanes between 3 & 4 and 7 & 8 are deliberately empty. (B) Longer exposure of the Rta western blot shown in (A) reveals that transfected Zta had induced Rta expression in 293-EBV-WT (but not 293-EBVΔRZ) cells that was not apparent on the shorter exposure.

We next infected 293 cells with either the parental Akata BACmid or EBVΔRZ (designated 293-EBV-WT and 293-EBVΔRZ, respectively). In 293-EBV-WT cells, transfection of Zta resulted in strong induction of early lytic BMRF1 expression, whereas BMRF1 induction by Rta was barely perceptible (Fig 1A, compare lanes 2 and 3). As expected, co-transfection of both Rta and Zta resulted in high level BMRF1 expression (Fig 1A, lane 4). Strikingly, in 293-EBVΔRZ, Zta alone did not induce any BMRF1 expression, but Rta alone resulted in low level BMRF1 expression. The combination of Rta and Zta resulted in synergistic BMRF1 expression as also observed in 293-EBV-WT cells. A longer exposure of the Rta western blot (Fig 1B) revealed that in 293-EBV-WT (but not 293-EBVΔRZ) cells, Zta transfection had also induced Rta expression. The apparent responsiveness of BMRF1 to Zta alone in this cell line was actually due to Zta expression combined with low level Rta expression. Therefore, it is only possible to examine the effect of Zta expression alone in the 293-EBVΔRZ background which reveals BMRF1 to be unresponsive to Zta, weakly upregulated by Rta alone, and highly expressed only when both Rta and Zta are present.

**Fig 2:**
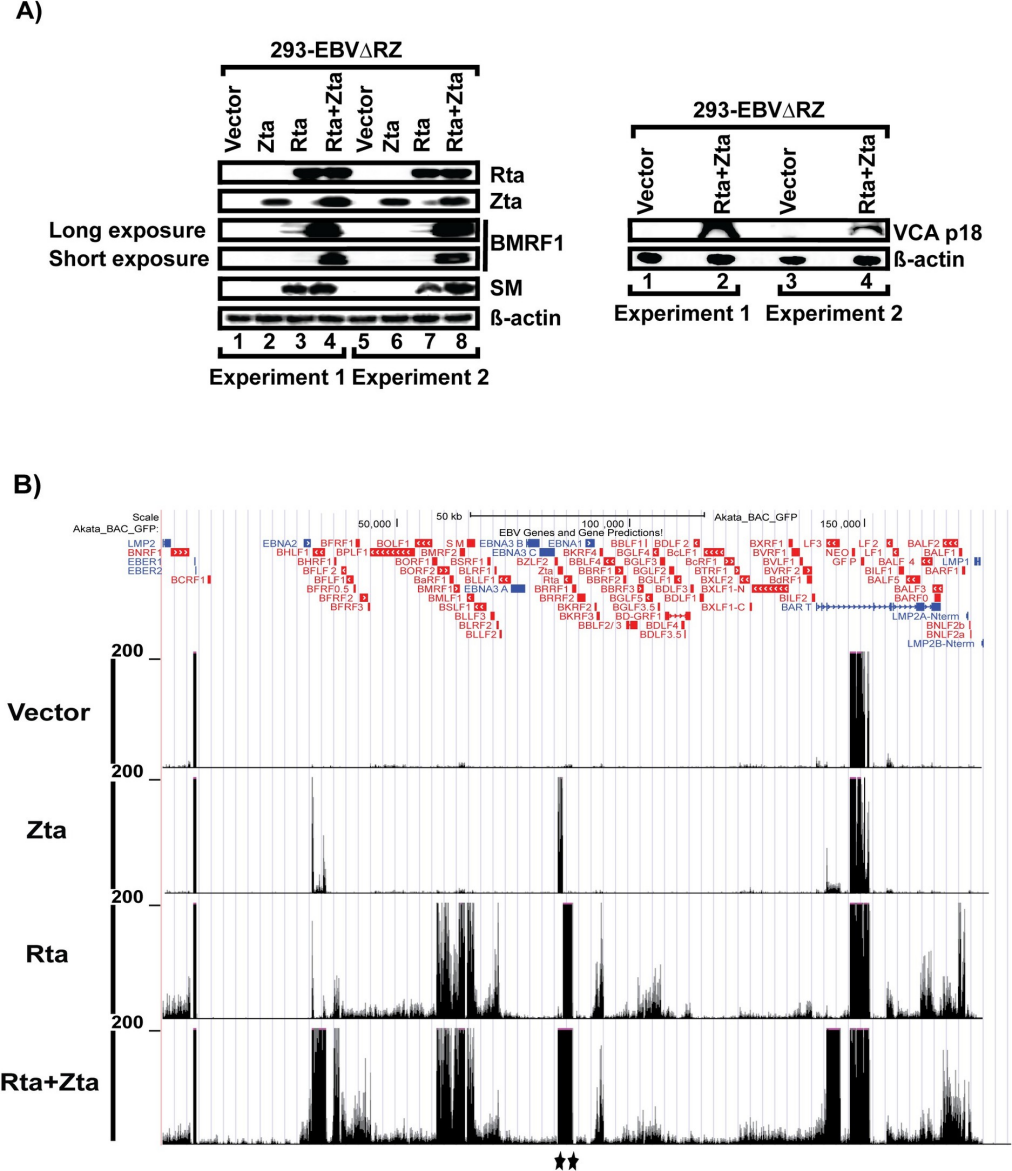
Rta and Zta dependence of EBV lytic gene expression in 293 cells. (A) Western blots showing the specified EBV or cell proteins from 293-EBVΔRZ cells transfected with vector only, Zta, Rta, or both (Rta+Zta). Data are two independent experiments from the same 293-EBVΔRZ clone. (B) UCSC genome browser images displaying RNA-seq data mapped to the EBV Akata_BAC_GFP genome. Each track corresponds to complementation with Rta and/or Zta as indicated on the left as described for (A). Black stars indicate reads arising from transfected Rta and/or Zta (i.e., not expressed from the EBV genome).

To characterize more fully the Rta versus Zta responsiveness of all lytic genes, we performed RNA-seq on trans-complemented 293-EBVΔRZ cells. For these experiments, we transfected 293-EBVΔRZ either with: empty vector, Zta, Rta, or Rta and Zta and harvested at RNA and protein lysates at 48 hours post-transfection. Each trans-complementation condition was assessed by western blotting for early (BMRF1 and SM) and late (VCAp18, encoded by BFRF3) gene expression (Fig 2A) which demonstrated that the full lytic cascade was activated by the combination of Rta and Zta. We then performed Illumina sequencing on ribosomal-depleted RNA libraries. As described in the methods, reads were aligned to the EBV genome using burrows-wheeler aligner (bwa). Mapped reads were converted into wiggle tracks and displayed using the UCSC genome browser (Fig 2B). In the absence of the Rta and Zta immediate early gene products, we observed only expression of the EBERs and the G418 and GFP selectable markers in EBVΔRZ infected cells. Consistent with prior results [37], this BACmid does not express the BART transcripts, likely due to transcriptional interference from the marker genes (Fig 2B, top track). Remarkably, transfection of Zta, resulted in activation of only two transcripts: LF3 and BHLF1 which are driven by the oriLyt promoters (Fig 2B, second track). By contrast, Rta transfection induced widespread activation of lytic promoters, resulting in expression of the majority of EBV early gene transcripts (Fig 2B, third track). As expected, Rta and Zta co-transfection resulted in transcription of all EBV lytic transcripts, including EBV late transcripts not detected with Rta alone (Fig 2B, bottom track).

### Rta is the primary driver of EBV lytic gene expression in NOKs-EBVΔRZ

**Fig 3:**
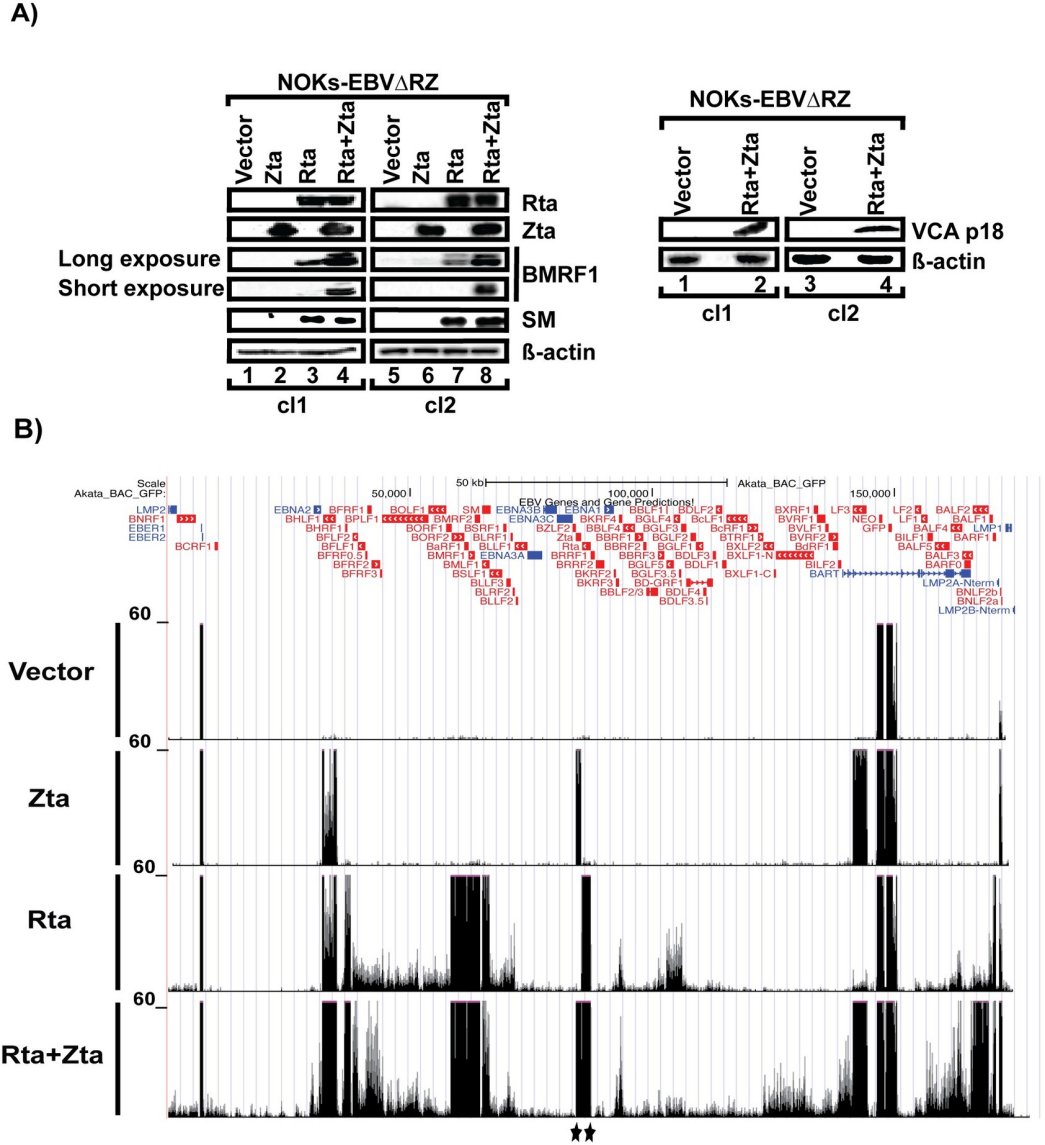
Rta and Zta dependence of EBV lytic gene expression in NOKs. (A) Western blots showing the specified EBV or cell proteins from NOKs-EBVΔRZ cells transfected with either Zta, Rta, Rta+Zta, or vector-only. The data are representative of two independent experiments. The data are from two different NOKs-EBVΔRZ clones and representative of two independent biologic replicates. (B) UCSC genome browser images displaying RNA-seq data mapped to the EBV Akata_BAC_GFP genome. Trans-complemented proteins are indicated to the left of each track and correspond to those used in (A). Black stars indicate reads arising from transfected Rta and/or Zta.

The inability of Zta to induce expression of any lytic transcripts other than those arising from the oriLyt promoters was unexpected. It was important to determine if this was unique to 293 cells or a more general phenomenon. We chose to examine this issue in the more physiologically relevant NOK cell line. NOKs exhibit normal keratinocyte morphology, retain the ability to differentiate when grown in raft cultures, and retain important anti-viral pathways including p53 and interferon signaling [38]. We first infected NOKs with the EBVΔRZ virus using the *E*. *coli* BM2710 invasive delivery and confirmed that the cell lines were intact for early and late gene expression upon trans-complementation with Zta + Rta (Fig 3A). We subsequently repeated our RNA-seq experiment, harvesting cells at 48 hours after trans-complementation with vector, Zta, Rta, or Rta + Zta. The lytic transcriptional landscape was similar to that seen in 293 cells (Fig 3B), except that we observed expression of the BNLF2a gene in vector transfected cells. BNLF2a is known to have a strongly keratinocyte-specific promoter and its latent expression has previously been reported by us and others [37,39]. Trans-complementation with Rta alone activated the expression of essentially all early genes, whereas Zta activation was limited to the oriLyt driven LF3 and BHLF1 transcripts and upregulated BNLF2a expression. Again, we observed late gene expression in response to Rta+Zta and strong, synergic activation of multiple early transcripts. Collectively, these results indicate that Rta is the primary driver of EBV early gene expression in these epithelial cell lines, but Zta is required for completion of the lytic cascade.

### Classification of EBV lytic genes based on their Rta and Zta dependencies

**Fig 4:**
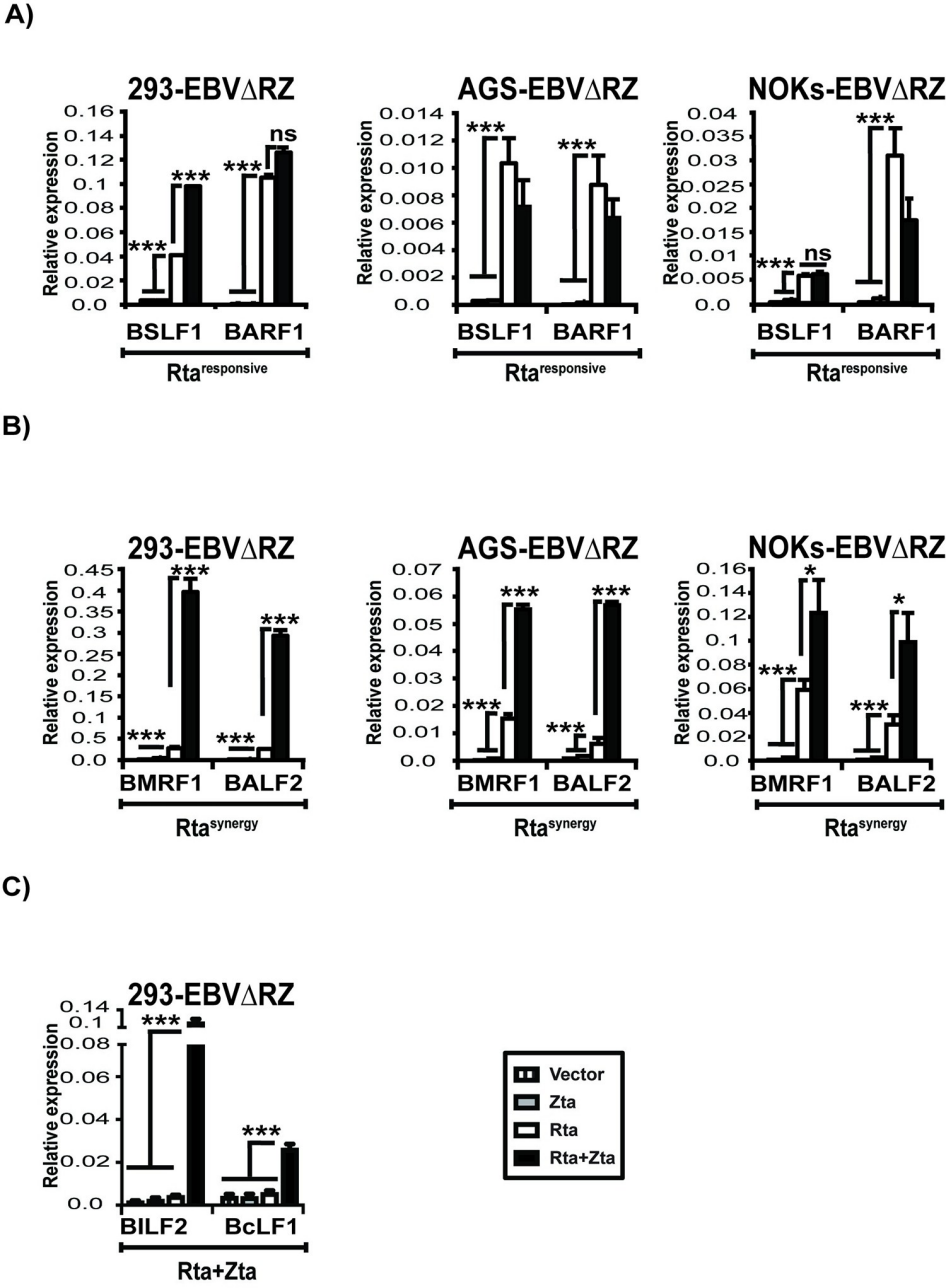
Classification of EBV lytic genes based on their Rta and Zta dependencies. Real time-qPCR data displaying the relative expression of various EBV lytic transcripts in 293-EBVΔRZ, AGS-EBVΔRZ and NOKs-EBVΔRZ. (A) Rta^responsive^ transcripts (BSLF1 and BARF1) depend only on Rta for expression. (B) Rta^synergy^ transcripts (BMRF1 and BALF2) are activated by Rta, but expressed at higher levels with Zta co-expression. (C) Rta+Zta transcripts (BcLF1 and BILF2) require both Rta and Zta transfection to be expressed. Results are expressed as mean values ± the standard error of the mean across two (AGS-EBVΔRZ, NOKs-EBVΔRZ) or one (293-EBVΔRZ) biological replicate. Data shown are representative of two independent experiments. Significant differences are indicated as follows: *P* ≤ 0.05 (*), *P* ≤ 0.01 (**), *P* ≤ 0.001 (***), P>0.05 (ns).

Our trans-complementation experiments suggested that EBV lytic genes, could be classified broadly into 3 groups based on their Rta and Zta dependencies. Some genes such as BSLF1 and BARF1 (Fig 4A) which required only Rta for their full activation were termed Rta^responsive^ genes. Other genes such as BMRF1 and BALF2 were activated by Rta but achieved full expression levels only in the presence of co-transfected Zta (Fig 4B) and were designated Rta^synergy^ genes. Finally, some genes such as BILF2 and BcLF1 required both Rta and Zta to be transcribed (Fig 4C). These were designated Rta+Zta genes and are best exemplified by late genes (which by definition require lytic DNA replication and therefore both Rta and Zta for their expression), but include some early and leaky genes as well (see below) [40]. These patterns were broadly consistent in the three different cell lines (293, AGS, and NOKs) that we examined, although the effect of Zta co-transfection was more pronounced in 293 cells than in either AGS or NOKs. In 293 cells, Rta^synergy^ genes were upregulated to a greater degree and some Rta^responsive^ genes (e.g., BSLF1) that experienced no increase with Zta co-expression in AGS and NOKs were upregulated by Zta co-expression in 293 cells, albeit to a much lesser degree than Rta^synergy^ genes such as BMRF1 and BALF2 (Fig 4A and 4B). Importantly, in all three cell lines, Zta alone did not activate transcription of any of these genes.

**Fig 5:**
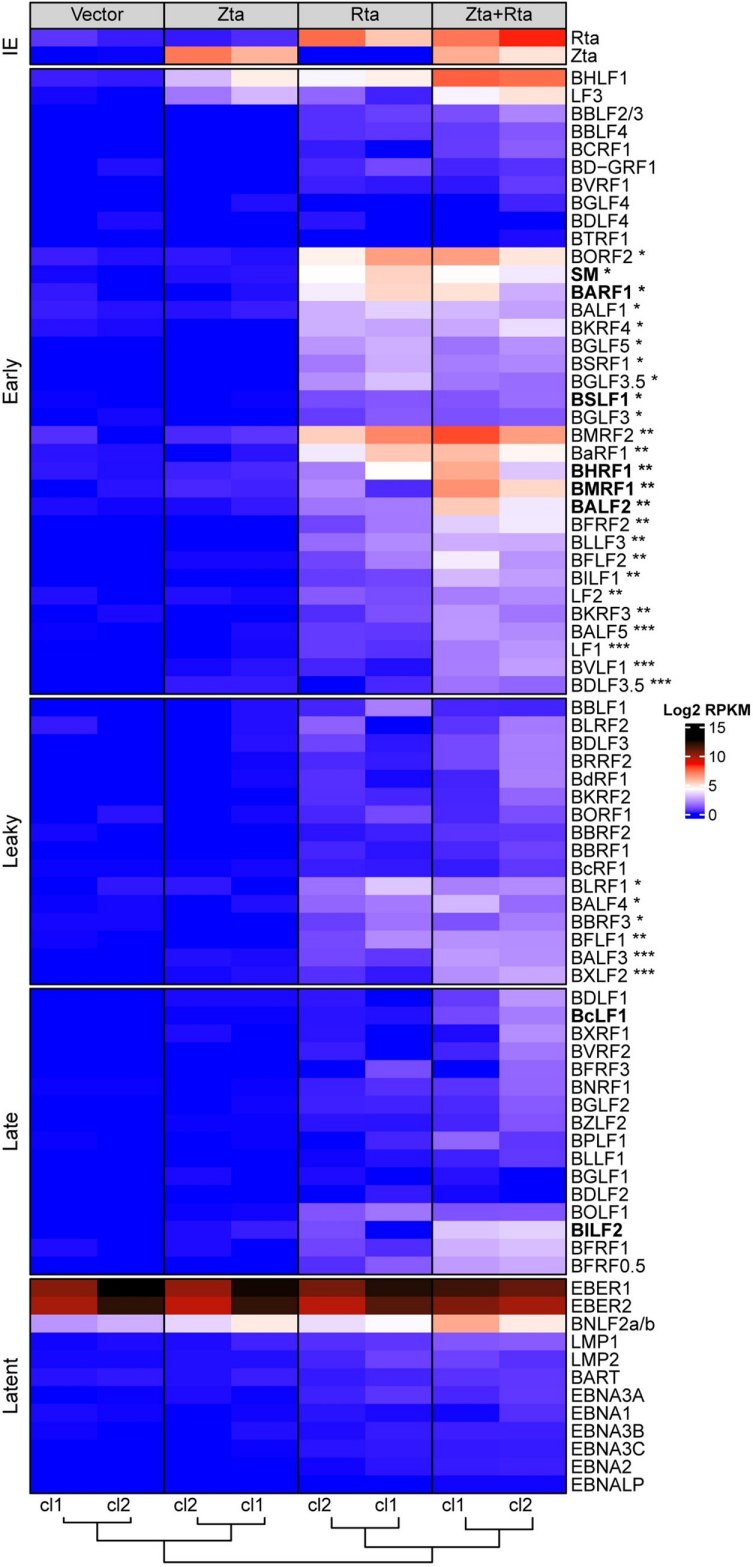
Responsiveness of EBV lytic transcriptome to Rta and Zta in NOKs. (A) Heatmap displaying expression levels of individual EBV genes derived from RNA-seq of two biological replicates of NOKs-EBVΔRZ (cl1, cl2) transfected either with vector, Zta, Rta, or Rta+Zta as indicated. Transcript names are indicated to the right and kinetic class to the left. For each gene, responsiveness to Rta and Zta is indicated as follows: Rta^responsive^ transcripts (*), Rta^synergy^ transcripts (**), Rta+Zta transcripts (***). Transcripts were quantified by log2 of the RPKM and color coded as indicated at bottom right. Transcripts that were subject to further analysis (Figs 4, 9 and 10) are shown in bold font.

In order to classify EBV lytic genes according to our schema of responsiveness (Rta^responsive^, Rta^synergy^, and Rta+Zta), we used our recently described UTS method [41] to quantify expression of each transcript in our different trans-complementation conditions. For each early or leaky transcript with sufficient depth to interpret (see materials and methods for details) we applied the following definitions: Genes that were only expressed in response to trans-complementation with both Rta and Zta were classified as Rta+Zta genes (Fig 5). Those achieving > 80% of their full expression with Rta alone were classified as Rta^responsive^, whereas those whose expression was ≤ 80% of their full expression with Rta alone were classified as Rta^synergy^ (Fig 5 and S1 Table).

### Zta actions at oriLyt and at methylated CpGs are dispensable for coactivation of early genes with Rta

**Fig 6:**
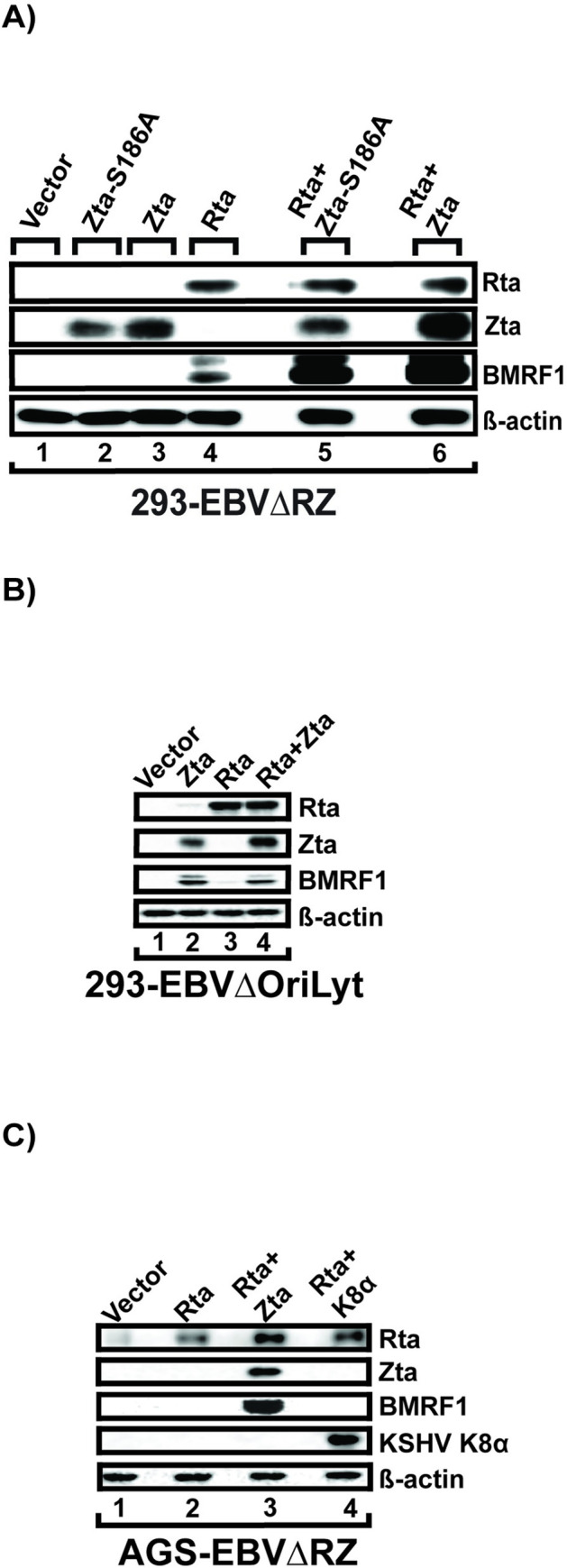
Characterization of Rta co-activation by Zta. (A) Western blots showing expression levels of the indicated EBV proteins in 293-EBVΔRZ trans-complemented with either Rta alone or Rta plus Zta-wt (Zta) or a methyl-DNA binding defective Zta mutant (Zta-S186A). Western blot images are representative of two independent experiments. (B) Western blot data from 293 cells infected with an EBV Bacmid (B95-8 strain) deleted for oriLyt transfected with vector control, Zta, Rta, or Rta and Zta and probed for the indicated proteins. (C) Western blots for the indicated proteins from AGS-EBVΔRZ transfected with Rta alone, Rta and Zta, or Rta and KHSV K8alpha.

By contrast to the widespread activation of early lytic promoters seen with Rta, Zta’s activity appeared to be restricted to activation of the oriLyt driven LF3 and BHLF1 transcripts and, in NOKs, upregulation of BNLF2a. We were interested in understanding how Zta could exert no transcriptional effects at these promoters in the context of the viral genome, yet strongly coactivate in the presence of Rta. Previous studies examining synergy between Rta and Zta have relied on artificial models, principally on reporter gene assays, which suggested that Zta alone could activate a variety of early lytic promoters [35,42,43]. Our EBVΔRZ system offered the opportunity to re-examine the mechanism of this synergy using the EBV genome as a readout. We hypothesized that Zta’s strong activation of the oriLyts (which contain AP-1 ZREs), but not early genes, was due to hypomethylation of CpG-containing ZREs in epithelial cells as previously observed [29]. Consistent with this, the Zta-S186A mutant which is defective for binding methylated CpG-ZREs [10] was able to synergistically activate BMRF1 expression comparably to Zta-WT (Fig 6A).

We next asked whether the oriLyt transcripts (BHLF1 and LF3) themselves could be playing a role in Zta’s ability to co-activate at Rta^synergy^ promoters. To address this, we used a previously described 293 cell line infected with an EBV BACmid mutant lacking both oriLyts (293-EBVΔoriLyt) [44]. Notably, the ΔoriLyt BACmid mutant is not deleted for BRLF1 or BZLF1 and we therefore cannot isolate the direct effects of Zta transfection from those due to induction of endogenous Rta. Thus, as we demonstrated in Fig 1, Zta alone “appeared” to induce full BMRF1 expression, due to Zta’s ability to also induce Rta expression from the EBV genome (Fig 6B, note low level expression of Rta in lane 2). Nevertheless, because full activation of BMRF1 expression was not observed with Rta alone, but did occur with Zta and Rta co-transfection (Fig 6B, lane 4), we were able to establish that the oriLyt transcripts are not required for Zta synergy.

Our finding that Zta’s principal role in epithelial cells is to support viral DNA replication mirrors the role of K8, its KSHV ortholog. We therefore wondered whether KSHV K8, could substitute for Zta in co-activation. We trans-complemented AGS-EBVΔRZ cells with a control vector, Rta with Zta expression plasmid (Rta+Zta) or KSHV K8 expression plasmid (Rta+KSHV K8 alpha) or Rta alone. As we observed earlier in 293 and NOK cells (Figs 2A and 3A), Rta alone resulted in weak BMRF1 expression. In contrast, Rta and Zta co-transfection induced full activation of BMRF1 expression, but KSHV K8 alpha co-transfection with Rta did not (Fig 6C). These results indicate that KSHV K8 is unable to substitute for Zta in co-activation.

We also used our trans-complementation approach to define Zta domains essential for co-activation. Zta mutants deleted for one activation domain (Zta-d27/53 or Zta-102/153) were competent for coactivation, but a Zta mutant defective for DNA binding (Zta-dbm1) could not coactivate BMRF1 or BHRF1 expression (S2A–S2C Fig). Collectively, these results suggest that Zta coactivation is occurring directly at lytic promoters and not via effects at oriLyt and that methylation of these lytic promoters is not required for Zta to synergize with Rta.

### Rta increases Zta binding to EBV early gene promoters

**Fig 7:**
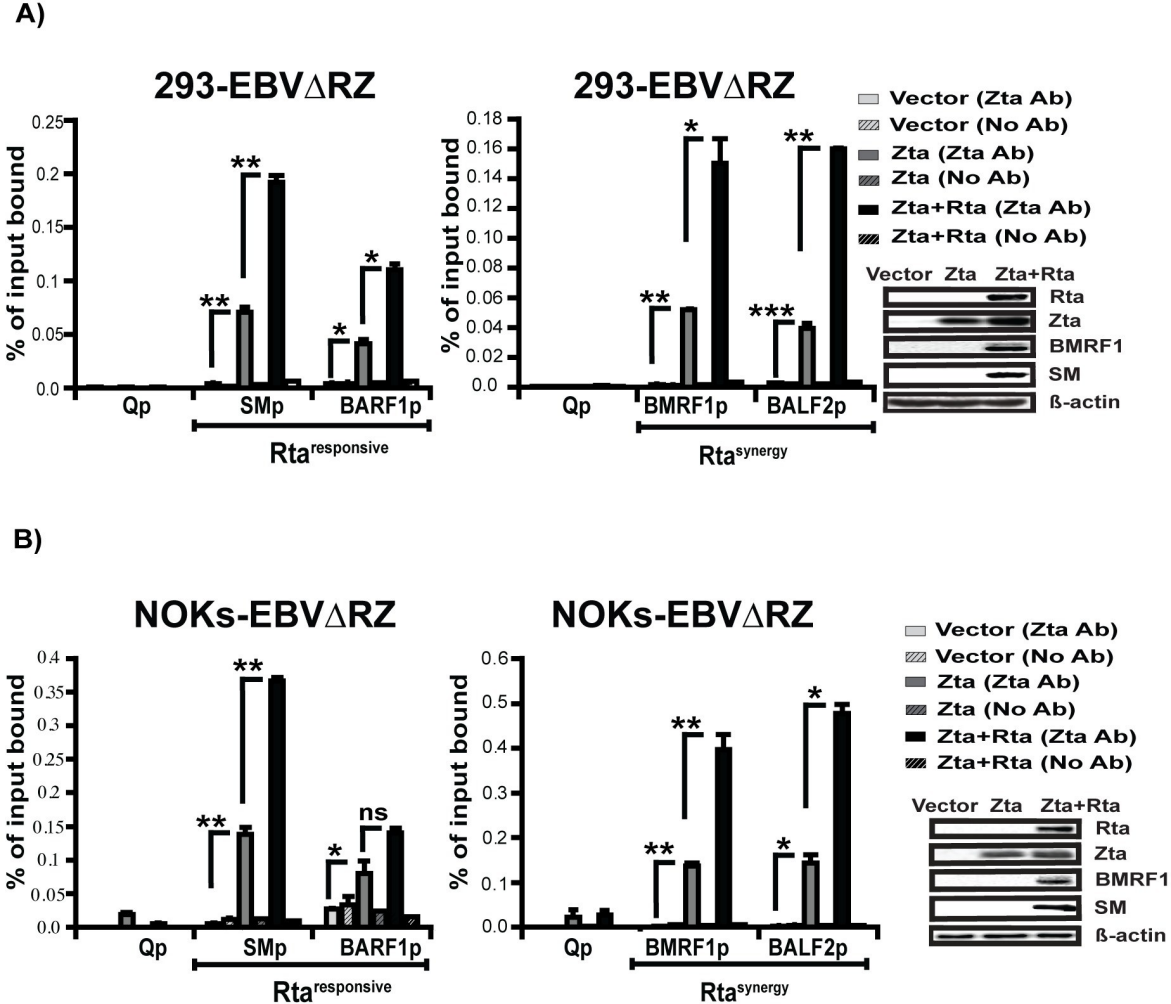
Rta increases Zta binding at Rta^responsive^ and Rta^synergy^ gene promoters. Chromatin immunoprecipitation and quantitative PCR (ChIP-qPCR) data for (A) 293-EBVΔRZ or (B) NOKs-EBVΔRZ showing Rta association with control (Qp), Rta^responsive^ (SMp and BARF1p) and Rta^synergy^ (BMRF1p and BALF2p) promoters. Cross-linked chromatin prepared from cells were trans-complemented with vector, Zta, or Zta and Rta as indicated. Cross-linked chromatin was immunoprecipitated with anti-Zta antibody (Zta Ab) or left untreated (No Ab) and quantified relative to input DNA. For each chromatin prep, western blots were performed (far right panels) to ensure early gene induction (BMRF1 and SM) and that equivalent amounts of Zta were expressed in the Zta and Zta+Rta conditions. Data shown in (A) are representative of two independent experiments and the NOKs ChIP (B) was performed once. Error bars indicate standard error of the mean and significant differences are indicated as follows: *P* ≤ 0.05 (*), *P* ≤ 0.01 (**), *P* ≤ 0.001 (***), P>0.05 (ns).

To understand why Zta was only able to activate most early gene transcripts in the presence of Rta, we asked whether the binding of Zta to early gene promoters was increased by Rta co-expression. These promoters included the SM, and BARF1 promoters (Rta^responsive^ genes), as well as the BMRF1 and BALF2 promoters (Rta^synergy^ genes). Prior to preparing chromatin samples for ChIP, we performed western blots to ensure induction of early gene expression and equivalent levels of Zta expression in the Zta versus Zta+Rta conditions (Fig 7A and 7B, far-right panels). Remarkably, in both 293 and NOKs, we observed Zta binding to both Rta^responsive^ (SMp and BARF1p, Fig 7A and 7B, dark gray bars, left panels) and Rta^synergy^ (BMRF1p and BALF2p, Fig 7A and 7B, dark gray bars, middle panels) promoters that was significantly above background. In addition, co-transfection of Rta produced a 2-4-fold increase in Zta binding at each of these promoters (Fig 7A and 7B, black bars). Although this increase in binding may be important for transcriptional synergy, the fact that it was observed at both Rta^responsive^ and Rta^synergy^ promoters leaves open the question of how promoter-specific synergy is achieved.

Collectively, these results demonstrate that Zta is binding to EBV early gene promoters in 293-EBVΔRZ and NOKs-EBVΔRZ cells, but that this does not result in their transcriptional activation in the absence of Rta. Although Rta enhances Zta binding to these promoters, this effect appears to be global and not specific to promoters at which Zta synergizes with Rta to activate early gene transcription.

### Zta does not selectively increase Rta binding to the Rta^synergy^ gene promoters

**Fig 8:**
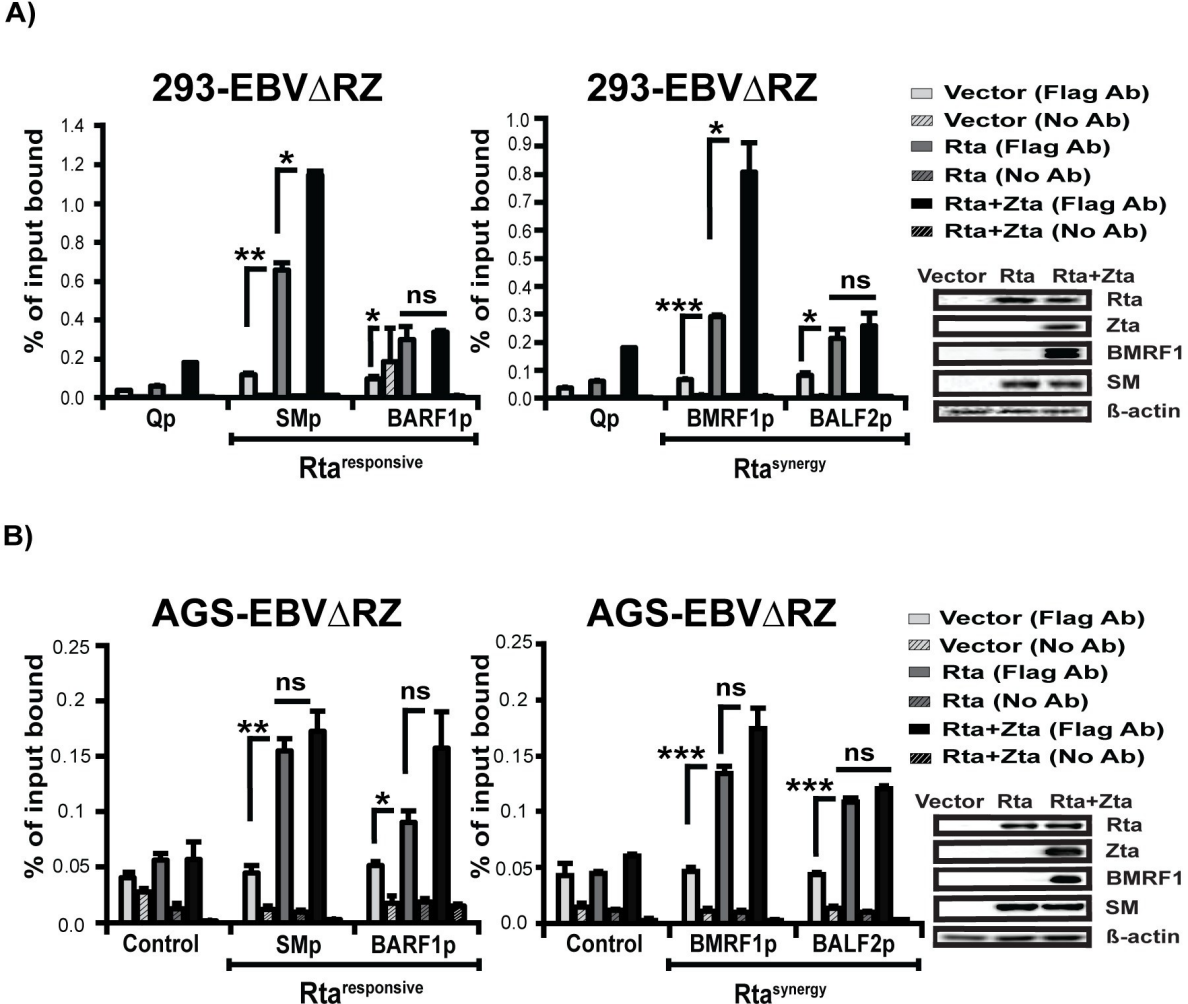
Zta increases Rta binding at Rta^responsive^ and Rta^synergy^ gene promoters. Chromatin Immunoprecipitation quantitative PCR (ChIP-qPCR) data from (A) 293-EBVΔRZ or (B) AGS-EBVΔRZ showing Zta association with control (Qp), Rta^responsive^ (SMp and BARF1p) and Rta^synergy^ (BMRF1p and BALF2p) promoters. Crosslinked-chromatin was prepared from cells trans-complemented with vector, Rta-flag (Rta) or Rta-flag and Zta (Rta+Zta) and immunoprecipitated using anti-Flag antibody (Flag Ab) or left untreated (No Ab) and quantified relative to input DNA. For each chromatin prep, western blots were performed (far right panels) to ensure early gene induction (BMRF1 and SM) and that equivalent amounts of Rta were expressed in the Rta and Zta+Rta conditions. Significant differences are indicated as follows: *P* ≤ 0.05 (*), *P* ≤ 0.01 (**), *P* ≤ 0.001 (***), P>0.05 (ns).

Since our results did not suggest that increased Zta binding was unique to Rta^synergy^ promoters, we next asked whether Zta co-expression resulted in increased Rta binding at these promoters. To examine this, we performed ChIP assays in 293-EBVΔRZ and AGS-EBVΔRZ cells transfected with Rta alone or Rta and Zta. As with our Zta ChIP assays, we first ensured early gene induction in transfected cells and that Rta expression levels were comparable between the two transfection conditions (Fig 8A and 8B, far-right panels). Our ChIP qPCR findings revealed that in 293-EBVΔRZ cells, Rta was associated with SM, BARF1, BMRF1, and BALF2 promoters as observed following Rta transfection (Fig 8A). However, Zta increased Rta binding (≤ 2-fold) across these tested promoters, compared to Rta alone (Fig 8A). We observed similar Rta binding to these four promoters in AGS-EBVΔRZ and again Zta co-expression was associated with a general trend of marginally increased Rta binding (Fig 8B). Importantly, despite these modest effects of Zta co-expression, we did not observe any differences at Rta^responsive^ versus Rta^synergy^ that would account for the dramatic effects of Zta co-expression at Rta^*synergy*^ promoters.

### Zta does not affect RNA stability of the early lytic Rta^synergy^ gene transcripts

**Fig 9:**
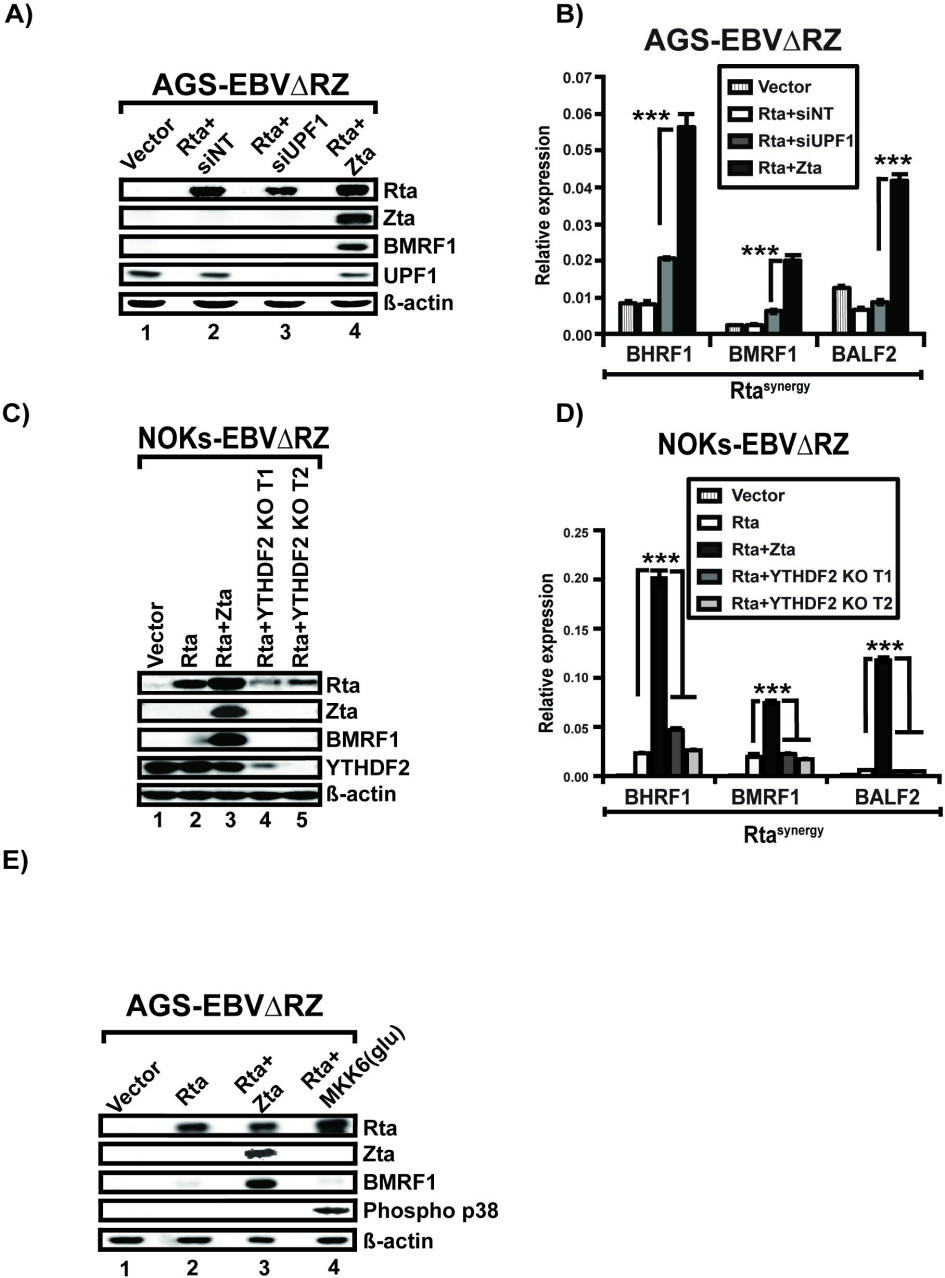
Zta does not affect RNA stability of the early lytic Rta^synergy^ transcripts. (A) Western blots of AGS-EBVΔRZ transfected with empty vector, Rta with non-targeting siRNA vector (Rta+siNT), Rta with UPF1 siRNA vector (Rta+siUPF1), or Rta with Zta (Rta+Zta) and probed with the indicated antibodies. (B) Real-time qPCR quantification from the same cells described in (A) showing expression levels of the indicated early Rta^synergy^ transcripts. (C) Western blot images of NOKs-EBVΔRZ cells harboring either wild-type YTHDF2 (lane 1–3) or knockout protein (lane 4–5) deleted by CRISPR/Cas9 using two different YTHDF2 sgRNA targets 1 and 2 (YTHDF2 KO T1 and YTHDF2 KO T2). Cells were transfected with Rta alone or Rta plus Zta (Rta+Z) where indicated, then probed for the indicated EBV proteins, YTHDF2, and beta actin control. (D) Real-time qPCR data of the trans-complemented NOKs-EBVΔRZ shown in (C) quantifying the expression levels of representative Rta^synergy^ transcripts as indicated. (E) Western blot of AGS-EBVΔRZ cells transfected with Rta alone, Rta and Zta, or Rta and constitutively active MKK6 mutant (MKK6(glu)) and probed for the indicated proteins. Significant differences are indicated as follows: *P* ≤ 0.05 (*), *P* ≤ 0.01 (**), *P* ≤ 0.001 (***), P>0.05 (ns).

Since our ChIP assays suggested that Rta/Zta synergy was not operating at the level of promoter binding, we considered the possibility that Zta was increasing the stability of Rta induced transcripts. Indeed, recent reports have shown that modulation of mRNA stability is a key mechanism by which gammaherpesvirus regulate the transition from latent infection to lytic replication [45–47]. Hypothesizing that Zta might interfere with the nonsense mediated decay (NMD) pathway to stabilize lytic transcripts, we tested whether NMD inhibition could substitute for Zta expression in increasing the levels of Rta^synergy^ transcripts. Initially we used an siRNA targeting the ATP-dependent RNA helicase upstream frameshift 1 (UPF1) [48], a critical effector of NMD. Despite robust UPF1 knockdown in AGS-EBVΔRZ cells, we did not observe any significant BMRF1 expression (Fig 9A, lanes 2 and 3) as occurred with Rta + Zta coexpression (Fig 9A, lane 4). Results were similar when we examined early mRNA levels by qPCR (Fig 9B, compare the dark gray bar to the black bar of each transcript). As a further test, we examined the ability of two small molecule inhibitors (NMDI-1 and NMDI-14), which target the interaction between the UPF1 and the NMD effectors SMG5 and SMG7, respectively. Treatment of AGS-EBVΔRZ cells with these inhibitors resulted in no appreciable increase in the expression of the early lytic Rta^synergy^ transcripts BHRF1 and BMRF1 (S3B Fig) or BMRF1 protein levels (S3A Fig) compared with Rta transfection alone.

In addition to NMD, several recent studies have demonstrated the importance of the m6A modification pathway in regulating EBV lytic transcript stability. In particular, the m6A reader protein YTHDF2 has been demonstrated to promote the decay of lytic mRNAs [49,50]. We therefore examined whether Zta could be stabilizing Rta^synergy^ transcripts through effects on YTHDF2. We used CRISPR/Cas9 to construct two different YTHDF2 knockouts in NOKs-EBVΔRZ cells. In each cell line, we observed no increase in BMRF1 induction by Rta compared to that seen in the parental NOKs-EBVΔRZ cells (Fig 9C). We also examined levels of the Rta^synergy^ transcripts BHRF1, BMRF1, and BALF2 in these cells and saw no increase in their expression in the YTHDF2 knockouts (Fig 9D). As expected Rta and Zta co-transfection resulted in full activation of all tested transcripts. Based on these results we conclude that Zta is not increasing Rta^synergy^ transcript levels through effects on the YTHDF2 m6A reader protein.

Finally, we examined the p38 mitogen-activated protein kinase (MAPK) pathway which can stabilize a wide range of mRNAs[51]. Multiple reports support a role for the p38 MAPK promoting lytic replication and because Zta promotes p38 activation [24,52–56], we tested whether p38 MAPK activation could substitute for Zta co-expression in promoting Rta^synergy^ transcription. Transfection of a constitutively active MKK6 mutant [57] resulted in p38 MAPK activity in AGS-EBVΔRZ cells as verified by phospho p38 blot (Fig 9E), but unlike Zta transfection, did not result in increased BMRF1 expression levels in the presence of Rta. In summary, we were unable to find any evidence that Zta was promoting increased levels of Rta^synergy^ transcripts through regulation of transcript stability.

### Zta increases RNA transcription of the early lytic Rta^synergy^ genes

**Fig 10:**
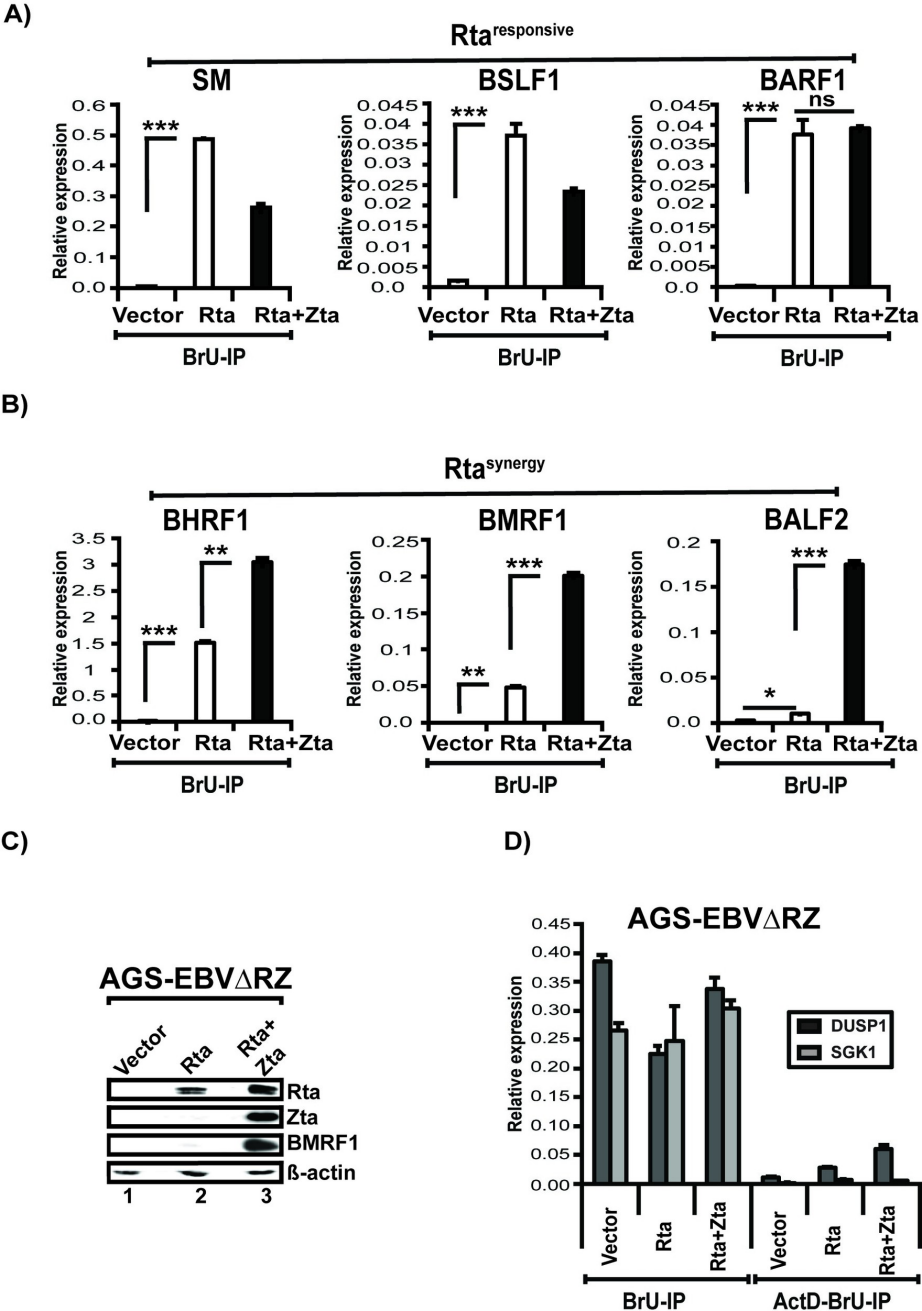
Zta increases nascent transcription of the early lytic Rta^synergy^ but not Rta^responsive^ gene transcripts. (A) BrU-labelling assay for nascent transcription of the SM, BSLF1, and BARF1 Rta^responsive^ transcripts. Forty-eight hours following transfection with vector, Rta, or Rta+Zta, AGS-EBVΔRZ cells were pulse-labelled with BrU for 1 hour, subjected to anti-BrU immunoprecipitation, and labelled transcripts assayed by quantitative RT-PCR. (B) Relative levels of nascent transcription of the BHRF1, BMRF1, and BALF2 Rta^synergy^ transcripts in AGS-EBVΔRZ cells examined by BrU-labelling assay. (C) Control western blot of AGS-EBVΔRZ cells used in panels A-B and probed for the indicated proteins. (D) Control BrU-assays performed in AGS-EBVΔRZ cells to demonstrate lack of BrU labelling of the Actinomycin-D (ActD) sensitive cellular transcripts DUSP1 and SGK1 in the presence of ActD. For all bar graphs, values are expressed relative to cellular labelled GAPDH mRNA and error bars indicate standard error of the mean. Data are representative of two independent experiments. Significant differences are indicated as follows: *P* ≤ 0.05 (*), *P* ≤ 0.01 (**), *P* ≤ 0.001 (***), P>0.05 (ns).

Although our ChIP experiments initially led us to discount the hypothesis that differences in transcription rates underpinned Rta + Zta synergy, the absence of differences in stability of Rta^synergy^ transcripts suggested synergy was due to transcription differences. As a more direct measure of the rate of transcription, we used 5-bromouridine (BrU) to label newly synthesized RNAs in AGS-EBVΔRZ cells at 48 hours post trans-complemention with Rta or Rta+Zta. We quantified BrU labelled mRNAs by RNA immunoprecipitation (IP) with anti 5-BrdU antibody followed by RT-qPCR for specific transcripts. Our results revealed that Zta co-transfection did not increase the rate of transcription of the SM, BSLF1, or the BARF1 Rta^responsive^ transcripts (Fig 10A). In contrast, we observed a marked increase in the BHRF1, BMRF1, and BALF2 Rta^synergy^ transcripts with Zta co-transfection relative to that induced by Rta alone (Fig 10B). Control western blots demonstrated that the levels of Rta expression in both conditions were well matched (Fig 10C). As an additional control, we performed our BrU-IP assay in cells treated with the transcription elongation inhibitor Actinomycin-D just prior to addition of BrU label. The BrU-IP assay readily detected the SGK1 and DUSP1 transcripts, but this signal was lost with Actinomycin-D treatment (Fig 10D). As SGK1 and DUSP1 have well characterized Actinomycin-D sensitive transcription [58] this result confirms that the BrU-IP assay is specific for measuring transcription of nascent RNAs. Collectively, these results indicate that Zta acts at the level of transcription to increase levels of the Rta^synergy^ transcripts induced by Rta.

## Discussion

In this manuscript, we have exploited an EBV mutant deleted for the IE gene locus to delineate the specific roles of Rta and Zta in early gene activation in epithelial cells. This approach was necessary because as we have shown (Fig 1), transfection of either Rta or Zta into cells infected with WT EBV produces confounding secondary effects due to induction of the other IE protein, even when its expression is not readily detected by western blotting. Our results are broadly consistent with prior reporter assays that have shown that Zta is relatively less important for early gene activation than Rta due to hypomethylation of early EBV promoters in epithelial cells. In those studies, Zta activated most unmethylated promoters 5-fold or less, but could activate some (including BMRF1 and BHRF1) over 100-fold [29]. However, our results, using native EBV early promoters in their genomic context, reveal a much starker dichotomy. Zta is not less important for activation of early promoters in epithelial cells, but rather Zta is unable to activate *any* protein coding genes (in 293 cells, Fig 2B) or activates *only one* protein coding gene (BNLF2a, in NOKs, Fig 3B) and functions principally to activate oriLyt transcription. Importantly, our results confirm the findings of Feederle et al. [28], that the EBV lytic cycle cannot be completed without both Rta and Zta. Our EBVΔRZ virus does, however, produce infectious virions when trans-complemented with both Rta and Zta (S1 Fig).

These results contribute to the growing evidence challenging Zta’s status as the principal conductor of the EBV lytic cascade. At least in epithelial cells, EBV replication control appears to be much more similar to KSHV than initially appreciated. In KSHV, the lytic cycle entry is controlled by the Rta ortholog ORF50 (KSHV RTA) and the Zta ortholog (K8) plays no role in early gene expression, functionally solely as a b-ZIP oriLyt binding protein required for DNA replication. This appears to be the arrangement in all rhadinoviruses implying this was the role of ORF50 in the common gammaherpesvirus ancestor. Gammaherpesviruses have evolved multiple mechanisms to repress expression of their ORF50 homologs, achieving exquisite control of entry into the lytic cycle. Recent work has shown that ORF50 transcripts, by including splice junctions downstream of the ORF50 stop codon are degraded by nonsense mediated decay that must be subverted to achieve lytic cycle entry [45,46]. In the case of EBV, further repression is achieved by extensive methylation of the Rta promoter in B lymphocytes, the site of latency, that requires binding of Zta to methylated response elements to overcome [10,11,13,15,16,29]. In this way, EBV appears to have placed its bZIP transcription factor at the top of lytic cascade, partially obscuring the essential role of Rta in early gene activation. Although these mechanisms are important for the EBV persistence strategy, it was important for us to bypass these regulatory mechanisms in order to directly examine the role played by each IE protein in activation of early gene promoters.

We initially anticipated, based on previous reporter assay data, that we would identify specific early genes that were upregulated by Rta or by Zta. Instead, our results show that Rta is the only direct activator of early gene transcription in epithelial cells. Some of these, such as SM are activated to very high levels by Rta alone–genes referred to here as Rta^*responsive*^. For other early genes such as BMRF1 and BALF2, Rta activates low level transcription and Zta functions as a coactivator. We refer to these as Rta^synergy^ genes. Such classification is not conceptually novel [28,30], but our study offers several advantages over prior attempts. First, by analyzing RNA-seq with our UTS read assignment method we simultaneously assessed the transcriptional dependence of nearly all lytic genes. Second, by using a genetic knock-out of the IE locus, we avoid secondary effects due to Rta and Zta inducing each other’s expression. Finally, because we are using the EBV genome as its own reporter, we expect to capture effects due to long-range enhancers and avoid artifacts introduced by transient reporter gene assays. It will be important to confirm our findings using other EBV strains and, as discussed below, assess these dependencies in B lymphocytes as well. Nevertheless, our results represent the most comprehensive assessment of the dependences upon Rta vs. Zta of EBV lytic genes published to date.

Why does Zta not directly activate lytic genes in epithelial cells? EBV genome hypomethylation is almost certainly a determining factor in Zta’s diminished role in early gene activation in epithelial cells. However, it is remarkable that our ChIP assays demonstrate Zta is binding to promoters, but fails to activate them. One interesting possibility is that that Zta’s effects when bound to methylated ZREs are qualitatively different than at unmethylated ZREs. For example, conformational changes induced by binding to methylated DNA may increase Zta’s ability to recruit HATs or RNA polymerase. Similar mechanisms have been invoked to explain differential effects of NF-kB transcription factors bound to different variants of its consensus sequence [59,60]. Alternatively, this lack of activation may be due to the need for B cell specific co-factors for Zta to directly activate transcription. This could include B cell transcription factors binding to early lytic promoters or proteins interacting with specific residues within the Zta activation domain that account for its cell type specific properties. The Zta activation domain is unusual in that it predominantly contains hydrophobic amino acids with aromatic and acidic residues embedded within glutamine and proline rich sequences. Previous work has suggested that the hydrophobic residues are relatively more important in HeLa and 293 cells, hinting at epithelial specific aspects of the Zta activation domain [61]. Finally, though we cannot rule out the possibility that Zta binding to hypomethylated early promoters in epithelial cells is merely below the threshold required for activation, its ability to activate the same promoters when unmethylated in reporter assays by 5–100 fold [29], favors the more nuanced mechanisms discussed above.

One notable exception to Zta’s inability to directly upregulate early transcripts was BNLF2a, which was only seen in NOKs. Interestingly, the ED-L2 promoter that drives BNLF2a expression is known to be activated by keratinocyte specific factors, particularly KLF4 [62]. In NOKs, BNLF2a is expressed at low levels in the absence of either Rta or Zta, consistent with its expression as a latent gene in some epithelial cells [37,39]. Thus, this apparent exception may prove the rule: Zta does not activate BNLF2a in keratinocytes, but rather synergizes with KLF4 to co-activate BNLF2a much the way it synergizes with Rta (i.e., BNLF2a may be a KLF4^synergy^ transcript).

How does Zta co-activate early gene expression with Rta in epithelial cells? Although synergy between Rta and Zta is well established [32–36,63], our EBVΔRZ virus allowed us to investigate it mechanistically in a highly physiologic system where the levels of Rta and Zta expression can be varied independently. Our ChIP assays demonstrated cooperative binding between Zta and Rta that may be mechanistically important for synergy; however, because we also observed this at Rta^responsive^ promoters, it does not account for the specific upregulation of Rta^synergy^ transcripts by Zta. We therefore examined whether Zta could be acting non-transcriptionally to stabilize Rta^synergy^ transcripts. Although NMD, m6A modification and the MAPK pathways have been implicated in EBV lytic transcript regulation, we found no evidence that these were involved in Zta’s ability to increase Rta^synergy^ transcript levels in epithelial cells. Instead, our BrU labelling experiments suggested that Zta is acting in concert with Rta to increase transcription of these genes, despite being unable to exert any transcriptional effects when expressed alone.

We performed ChIP assays in AGS-EBVΔRZ cells to examine the effect of Zta co-transfection at various Rta^responsive^ and Rta^synergy^ promoters. We found no evidence that Zta was increasing levels of the activating H3K27ac or H3K9ac histone marks at Rta^synergy^ promoters (S4A and S4B Fig). This is consistent with previous work has shown that Zta does not robustly promote H3K9 acetylation [29,64] in epithelial cells, despite the shared ability to interact with histone acetylases. Our ChIPs also demonstrated no differences in RNA polymerase II occupancy due to Zta co-expression above that seen with Rta alone at either Rta^responsive^ or Rta^synergy^ promoters (S4C Fig). These results leave open the possibility that Zta is acting at Rta^synergy^ promoters to relieve a transcriptional pause. This is increasingly appreciated as a critical regulatory step in gene expression. Much less is known about why some genes are subject to this control whereas others are not. But in this model the Rta^responsive^ genes are not subject to pausing and thus do not require Zta to achieve full expression.

An alternative mechanism by which Zta could promote early gene transcription without direct transactivation is by acting as a pioneer factor. Indeed, the clustering of many of the synergy transcripts in the genome (see S5 Fig) suggests that they may share a common epigenetic context. Our ChIP assays demonstrate that there is sufficient accessibility for Rta to bind to Rta^synergy^ promoters in the absence of Zta. Nevertheless, Zta’s ability to recruit chromatin remodelers such as INO80 to may overcome chromatin barriers impacting transcription at Rta^synergy^ promoters that are not impinging Rta’s actions at Rta^responsive^ ones. In support of this Schaeffner et al. [65], found that knockdown of INO80 impaired expression of some early transcripts like BMRF1 (an Rta^synergy^ transcript), BNLF2a (a likely KLF4^synergy^ transcript), but not others such as BBLF4 (an Rta^responsive^ one).

An important unanswered question is which early genes will EBVΔRZ reveal to be regulated by Rta versus Zta in B lymphocytes. Since the role played by each cell type in the EBV life cycle is different, it would not be surprising to find significant differences between the two cell types. Lytic replication in B lymphocytes serves to reactivate EBV from latency and Zta’s dependence upon viral genome methylation prevents activation of the lytic cascade until after latency is established [15]. In contrast, lytic replication of epithelial cells serves amplify the quantity of EBV released and the lytic cascade can proceed directly without the requirement for extensive viral genome methylation [29,66,67]. Zta is clearly the dominant activator of the lytic cascade in B lymphocytes; however, much of this derives from its unique ability to robustly activate the Rta promoter when methylated. Studies using Raji cells, where Rta is unable to induce detectable amounts of Zta, argue that Rta alone is sufficient to activate several lytic promoters including BMRF1, BHRF1, and BLRF2 [30]. To our knowledge, the Zta responsiveness of lytic genes using the BRLF1-KO virus has only been examined for a few genes in epithelial cells and not characterized in B lymphocytes [28,68]. Although reporter assays suggest that Zta plays a significant role in the activation of early promoters in B lymphocytes [32,43,69], we have demonstrated that crucial differences can exist between reporter assay predictions and actual lytic gene expression from the EBV genome. We are currently deriving LCLs transformed by our EBVΔRZ virus to characterize the dependence of each early promoter upon Rta versus Zta during reactivation from B lymphocyte latent infection.

## Materials and methods

### Cell lines

The normal oral keratinocyte (NOKs) cell line (a generous gift from Karl Munger) was derived by telomerase immortalization of human gingival tissue [38]. AGS is an EBV-negative human gastric adenocarcinoma cell line that was obtained from the ATCC. HEK293 is an epithelial cell line derived by immortalizing human embryonic kidney tissue with adenovirus type 5 DNA. HEK293 (referred to as 293 in the text) was obtained from ATCC and is known to express neuronal markers and may be of adrenal origin [70]. HeLa is an HPV18 infected cervical carcinoma cell line obtained from Bill Sugden. NOKs were cultured in keratinocyte serum-free medium (KSFM) (Life Technologies, Inc.), supplemented with epidermal growth factor and bovine pituitary extract. All other cell lines were maintained in Dulbecco’s Modified Eagle Medium (Thermo Fisher Scientific, US) supplemented with 10% fetal bovine serum.

### Plasmids and BAC mutagenesis

EBVΔRZ was previously described [37,71]. pSG5-Rta and pSG5-Zta (aka pSVNaeZ) expression vectors have been previously described [72,73]. The Zta-d27/53, Zta-d102/153, and the Zta-dbm1 construct were kind gifts of Erik Flemington [74,75]. The Rta-Flag expression vector [76] was a kind gift of Lori Frappier.

### Derivation of EBVΔRZ infected epithelial cell lines

The EBVΔRZ Bacmid was delivered to cultured epithelial cells using BM2710 invasive E. coli as previously described [44]. Briefly, EBVΔRZ was electroporated using a 0.1 cm gap cuvette (1.5 kV, 200 Ohms, 25 μF) into BM2710 E. coli and selected with Kanamycin. BM2710 E. coli containing EBVΔRZ were then used to infect EBV-negative epithelial cells by co-incubation for 2 hours (approximately 25 bacteria per cell). Cell lines were derived by single-cell cloning and, when possible, screened for ability to complete the lytic cascade by immunoblotting for viral late protein VCAp18 upon transfection with Rta and Zta. EBVΔRZ-positive cells were selected and maintained with 50–600 μg/ml of G418.

### Cell transfections

Cells were transfected with expression vectors using Lipofectamine 2000 (Invitrogen) following the manufacturer’s protocol. Plasmid DNA extracted using CompactPrep Plasmid Midi Kit (QIAGEN) according to the manufacturer’s instructions. Approximately 7x10^5^ cells in log phase were transfected either with 0.5 μg control expression plasmid, Zta expression vector, Rta expression vector or 0.25 μg Rta plus 0.25 μg Zta expressions vectors for 48–72 hours.

### Western blot analysis

Cells were washed with phosphate buffered saline (PBS) and lysed with RIPA buffer (50 mM Tris HCL (pH 8.0), 150 mM NaCl, 1% NP-40 or TritonX-100, 0.5% Sodium Deoxycholate and 0.1% SDS) by incubation on ice for 30 min. Whole-cell lysates (WCL) were collected by centrifugation at 13,000 rpm for 20 min at 4°C. Protein concentrations were quantified by Coomassie (Bradford) assay kit (Thermo Scientific catalog #23200) as described by the manufacturer. Protein lysates were separated with SDS-PAGE, transferred to nitrocellulose membrane (cytiva catalog #10600007 #10600006), and incubated with primary antibody (1:200 anti-Zta Santa Cruz catalog #sc-53904, 1:250 anti-Rta a rabbit polyclonal antibody directed against the R peptide sequence EDPDEETSSQAVKALREMAD, 1:1000 anti-VCA p18 Thermo Fisher Scientific catalog #PA1-73003, 1:1000 anti-BMRF1 Millipore catalog #MAB8186, 1:200 anti-ß-actin Santa Cruz catalog # sc-47778, 1:800 anti-SM a kind gift from Sankar Swaminathan of the University of Utah, 1:1000 anti-phospho p38 MAPK Cell Signaling Technologies catalog #4511T, 1:1000 anti-UPF1 Cell Signaling Technologies catalog #12040S, 1:1000 anti-STREP abcam catalog #76950, and 1:10000 anti-YTHDF2 Proteinteck catalog #24744-1-AP), diluted in TBS with 5% skimmed milk at 4°C overnight. The membranes were then washed in TBS with 0.1% Tween-20 and incubated with secondary HRP-conjugated antibody (1:10000, mouse Thermo Fisher Scientific catalog #A24524), or (1:3000, rabbit, Thermo Fisher Scientific catalog #A10547) for 1 hour at room temperature. The membrane was washed again and developed with chemiluminescence reagent (Pierce ECL Western Thermo Fisher Scientific catalog #32106). Western blots were visualized and analyzed on Bio-Rad ChemiDoc Imager (Bio-Rad).

### RNA isolation and real time-quantitative polymerase chain reaction (RT-qPCR)

RNA was isolated from between 1-5x10^6^ transfected cells. Cells were harvested and lysed in Trizol reagent (Invitrogen). Two hundred microliters of chloroform were added to the lysed cells and centrifuged for 15 min at 15000 rpm, and isolated using isopropanol. The isolated RNA was then washed with 70% ethanol and dissolved in sterile water. The RNA was then treated with DNase (Thermo Fisher) following the manufacturer’s protocol. Isolated RNA (0.5–1μg) was used to make cDNA using GoScript Reverse Transcription Mix, and Random Primers kit (Promega) following the manufacturer’s protocol. Next cDNA was quantified using RT-qPCR and SYBR Green Real-Time PCR Master Mix (Biorad). Primers used in RT-qPCR in this study are in S2 Table. All quantified transcripts were expressed and graphed relative to cellular glyceraldehyde-3-phosphate dehydrogenase (GAPDH) expression, with error bars indicating standard error of the mean (SEM).

### Chromatin immunoprecipitation quantitative polymerase chain reaction (ChIP qPCR)

ChIP qPCR was done as described before [77]. Briefly, 1x10^7^ cells were transfected either with control expression plasmid, Zta expression vector, Rta expression vector or Zta plus Rta expression vectors for 24–48 hours, were harvested and cross-linked in 1% formaldehyde for 10 min at room temperature. This reaction was stopped by addition of 0.125 M glycine at room temperature for 10 min. Samples were then washed with 1X (PBS) and centrifugation at 4° C. These cells were lysed in 1 ml of cell lysis buffer (10 mM Tris HCl pH 8.0, 10 mM NaCl and 0.2% NP-40) for 10 min on ice. Collected nuclei were then lysed in 0.3 ml of nuclei lysis buffer (50 mM Tris HCL pH 8.0, 10 mM EDTA pH 8.0 and 1% SDS) for 10 min on ice. For shearing the DNA, the cell lysate was sonicated for 30 sec ON/ 90 sec OFF for 4–6 cycles using Fisher Scientific Sonic Dismembrator Model 100 sonicator setting 3. Of each sonicated sample, about 1 X 10^6^ (~25 μg chromatin) was immunoprecipitated with antibodies of interest [anti-Zta (Santa Cruz, catalog #sc-53904, 1 μg per ChIP), anti-Flag M2 magnetic beads (Sigma Aldrich, catalog #M8823), anti-Histone H3K27ac (Active Motif, catalog #39133, 10 μg per ChIP), anti-Histone H3K9ac (abcam, catalog #ab4441, 2 μg for 10^6^ cells), or anti-RNA polymerase II (abcam, catalog #ab5095, 4 μg for 10^6^ cells)] at 4° C overnight. The samples were then washed in a low salt wash buffer (20 mM Tris HCL pH 8.0, 2 mM EDTA, 150 mM NaCl, 0.1% Triton X-100, and 0.1% SDS). Then subsequently in a high salt wash buffer (20 mM Tris HCL pH 8.0, 2 mM EDTA, 500 mM NaCl, 0.1% Triton X-100, and 0.1% SDS). Later, in LiCl wash buffer (10 mM Tris HCL pH 8.0, 1 mM EDTA, 0.25 mM LiCl, 1% Deoxycholic acid and 1% NP-40 in addition to 2 washes in TE buffer. All washes were done at 4°C for 15 min. All lysis and wash buffers contained protease inhibitor cocktail (Roche, Germany, catalog #11836170001). Finally, these samples were eluted in elution buffer (0.1 M NaHCO3 and 1% SDS) at 65° C for 30 min, and then reverse cross-linked by NaCl (0.3 M) done at 65° C in the presence of RNase A (1 mg/ml). RNase A was inactivated by proteinase K (10 mg/ml) for 30 min at 65° C. Proteinase K (10 mg/ml) was inactivated at 65° C for 10 min. DNA-protein complexes were then phenol/ chloroform-extracted, ethanol precipitated and dissolved in TE buffer. Then quantified by qPCR using SYBR Green Real-Time PCR Master Mix (Biorad). Primers used in ChIP-qPCR in this study are in listed S3 Table.

### RNA-seq analysis

Cell pellets were harvested in Trizol and RNA was isolated according to the manufacturer instructions. RNA libraries were constructed using the Illumina TruSeq Stranded Total RNA LT kit as previously described [37] and sequenced on an Illumina HiSeq x 101 cycles at the University of Wisconsin-Madison Biotechnology Center. This RNA-seq data has been deposited at NIH SRA under accession number PRJNA850444.

Resulting reads were mapped to the Akata_BAC_GFP genome [37] using STAR (v2.7.6a) with default settings [78]. Reads were assigned to EBV genes using with mmquant and UTS using default settings [41,79] and a previously described EBV1 annotation file [41] except that the LMP2A and LMP2B genes were considered as a single gene whose expression level was calculated based on sequencing depth of the common exons (2–9). The heatmap in Fig 5 was generated using the ComplexHeatmap (v2.8.0) R package [80]. Specifically, EBV gene expression levels were quantified as log2-transformed RPKM values with a pseudo count of 1. Columns were clustered by k-means (n = 4) and rows by the Euclidean distance method within each subgroup of viral gene expression kinetics [40].

### Classification of Rta and Zta responsiveness of EBV lytic genes

We classified the Rta and Zta responsiveness of early and leaky genes according to the following schema. Genes expressed below 1 RPKM in the Rta+Zta condition were not classified. Genes expressed below 1.5 RPKM in the Rta condition were classified as requiring both Rta and Zta (Rta+Zta). Genes expressed at or above 1.5 RPKM in the Rta condition subdivided based on the ratio of their expression in the Rta versus Rta+Zta conditions. Those with [Rta]/[Rta+Zta] expression ratio > 0.8 were classified as Rta^responsive^, whereas those with [Rta]/[Rta+Zta] expression ratio ≤ 0.8 were classified as Rta^synergy^.

### CRISPR/ Cas9 knockout of YTHDF2

To knockout the m6A reader protein YTHDF2 of NOKs-EBVΔRZ cells, we used a previously described oriP vector to express both Cas9 and the sgRNA [81]. The sgRNA used were previously described by Zhang et al. [49], including YTHDF2 KO T1 (forward 5-caccAGTTACTACAGTCCCTCCAT-3 and reverse 5-aaacATGGAGGGACTGTAGTAACT-3), and YTHDF2 KO T2 (forward 5-caccGTCCATTACTAGTAACATCG-3 and reversed 5-aaacCGATGTTACTAGTAATGGAC-3). CRISPR/Cas9 plasmids were then transfected into NOKs-EBVΔRZ cells and maintained by Blasticidin selection at final concentration of 1.5 μg/ml.

### BrU labelling of host and EBV lytic transcripts

To study the RNA synthesis of EBV early lytic transcripts, we metabolically labeled newly synthesized RNAs with 5-bromouridine (BrU) followed by immunoprecipitation (IP) as previously described [82]. Briefly, 48 hours after transfection of AGS-EBVΔRZ cells with vector, Rta, or Rta+Zta, cells were treated with 5 μg/ml Actinomycin D (ActD) (Sigma Aldrich Inc catalog #A1410) for 30 min or left untreated. Next, all cells were labelled with 2 mM BrU (Fisher Scientific Company LLC, catalog AAA1850701) for 1 hour, followed by three washes with cold PBS. RNA was then isolated using RNeasy mini kit (QIAGEN) using the manufacturer’s protocol. Equal amounts of RNAs were immunoprecipitated using mouse anti-BrdU (BD Pharmingen catalog #555627, 1.25 μg per sample) antibody then quantified by RT-qPCR analysis as described above.

### Data analysis

RT-qPCR data was analyzed as follows. We calculated the ΔCt value for each gene (using 2–3 technical replicates) and normalized this to GAPDH by subtracting the GAPDH ΔCt value to obtain ΔΔCt. Relative expression (RE) level was calculated using the formula: RE = 2^^-ΔΔCt^. Mean relative expression levels were calculated combining technical and biological replicates and error bars graphed to indicate the standard error of the mean (SEM). *P* values were calculated by student’s-t-test with a two-tail distribution.

For ChIP-qPCR data, we calculated the ΔCt value for each target locus (using 2–3 technical replicates) and normalized this by subtracting ΔCt value obtained for the input chromatin (normalized to account for the fact that the input sample was 10% of the amount of chromatin used in each ChIP assay [i.e., by subtracting log_2_10 from the ΔCt value]) to obtain ΔΔCt*. Percent bound (PB) was calculated using the formula PB = 100*2^^-ΔΔCt^*. Mean percent binding was calculated by combining technical and biological replicates and error bars graphed to indicate the standard error of the mean (SEM). *P* values were calculated by student’s-t-test with a two-tail distribution.

## Supporting information

S1 Fig EBVΔRZ produces infectious virions. Fluorescent micrographs (top panels) and bright field images (bottom panels) of EBV-negative Akata Burkitt lymphoma (BL) cells either infected or uninfected with EBVΔRZ virions. Virions were produced from EBVΔRZ in infected HeLa cells transcomplemened with Rta and Zta. Supernatants harvested 96 hours later and passed through a 0.8 μm-pore filter to remove cellular debris. EBV-negative Akata BL cells (2X10^6^) were then infected with this supernatant for 2 hr at 37˚C. Images were obtained forty-eight-hours post-infection. green fluorescent protein (GFP) signals derive from a reporter cassette in the EBVΔRZ virus.
(TIF)

S2 Fig Characterization of Rta co-activation by Zta. (A) Western blots for the indicated proteins from NOKs-EBVΔRZ trans-complemented with Rta alone and Rta and one of the following Zta expression constructs: wild-type Zta (Zta wt) HA-tagged Zta (Zta HA wt), and Zta K178E, R179E, Y180L (Zta-dbma1)—a ZRE-binding-defective mutant. Mutant Zta-d27/53 and Zta-d102/153 are two Zta activation domain mutants. RT-qPCR data displaying the expression of BMRF1 (B) and BHRF1 (C) in NOKs-EBVΔRZ trans-complemented as described for (A). Significant differences are indicated as follows: *P* ≤ 0.05 (*), *P* ≤ 0.01 (**), *P* ≤ 0.001 (***), P>0.05 (ns).
(TIF)

S3 Fig Inhibition of NMD pathway does not substitute for Zta in upregulation of early lytic Rta^synergy^ transcripts. (A) Western blot of AGS-EBVΔRZ cells transfected with Rta or Rta plus Zta, and treated with the nonsense mediated decay inhibitors (NMDI-1 or NMDI-14) where indicated, then probed for the indicated EBV proteins, UPF1, and beta actin control. (B) Real-time qPCR data of the same cells described in (A) quantifying expression of two representative Rta^synergy^ transcripts (BHRF1 and BMRF1). Significant differences are indicated as follows: *P* ≤ 0.05 (*), *P* ≤ 0.01 (**), *P* ≤ 0.001 (***), P>0.05 (ns).
(TIF)

S4 Fig Zta does not increase levels of activating histone marks or RNA polymerase II occupancy at the early lytic Rta^synergy^ promoters. Chromatin immunoprecipitation assay for histone H3K27ac (A), H3K9ac (B), and RNA polymerase II (C) at the indicated EBV early promoters and a region of the EBV genome devoid of Rta binding (control) measured by quantitative PCR (ChIP-qPCR) in AGS-EBVΔRZ cells trans-complemented with Rta alone (dark gray bars) or with Rta and Zta (black bars). The qPCR data is reported as a percentage of the input sample with error bars indicating standard error of the mean. Chromatin samples used in (A) are from the same transfected cells shown in Fig 8B. Western blotting (middle right panel) was performed to ensure early gene induction (BMRF1 and SM) and equivalent expression of Rta in the Rta versus Rta+Zta conditions before preparing chromatin samples for the experiments done in (B) and (C). Significant differences are indicated as follows: *P* ≤ 0.05 (*), *P* ≤ 0.01 (**), *P* ≤ 0.001 (***), P>0.05 (ns).
(TIF)

S5 Fig Localization of Rta^responsive^, Rta^synergy^, and Rta+Zta early and leaky transcripts with the EBV genome. UCSC genome browser image highlighting the locations of early and leaky lytic transcript locations color coded by their Rta and Zta responsiveness. Rta^responsive^ transcripts are shown in pink, Rta^synergy^ transcripts in light purple, and Rta+Zta in dark purple.
(TIF)

S1 Table Classification of EBV lytic genes based on their dependencies on Zta and Rta in NOKs. Shown are the early and leaky transcripts whose responsiveness to Rta and Zta could be classified from our RNA-seq data from NOKs-EBVΔRZ (see Fig 5 for additional details). Note that all late genes are classified as Rta + Zta since they are by definition dependent on DNA replication which itself requires Rta + Zta.
(XLSX)

S2 Table List of primers used for reverse transcription quantitative PCR analysis. Forward and Reverse primer pairs are listed for each EBV or host transcript measured by RT-qPCR.
(XLSX)

S3 Table List of primers used for ChIP quantitative PCR analysis. Forward and Reverse primer pairs are listed for each EBV promoter examined by ChIP-qPCR as well as a region of the EBV genome not bound by Rta or Zta (control).
(XLSX)
