# Supplementary figures and images for: Rta is the principal activator of Epstein-Barr virus epithelial lytic transcription
Source: PLoS Pathog. 2022 Sep 29;18(9):e1010886. doi: 10.1371/journal.ppat.1010886 (PMC9553042; doi:10.1371/journal.ppat.1010886)

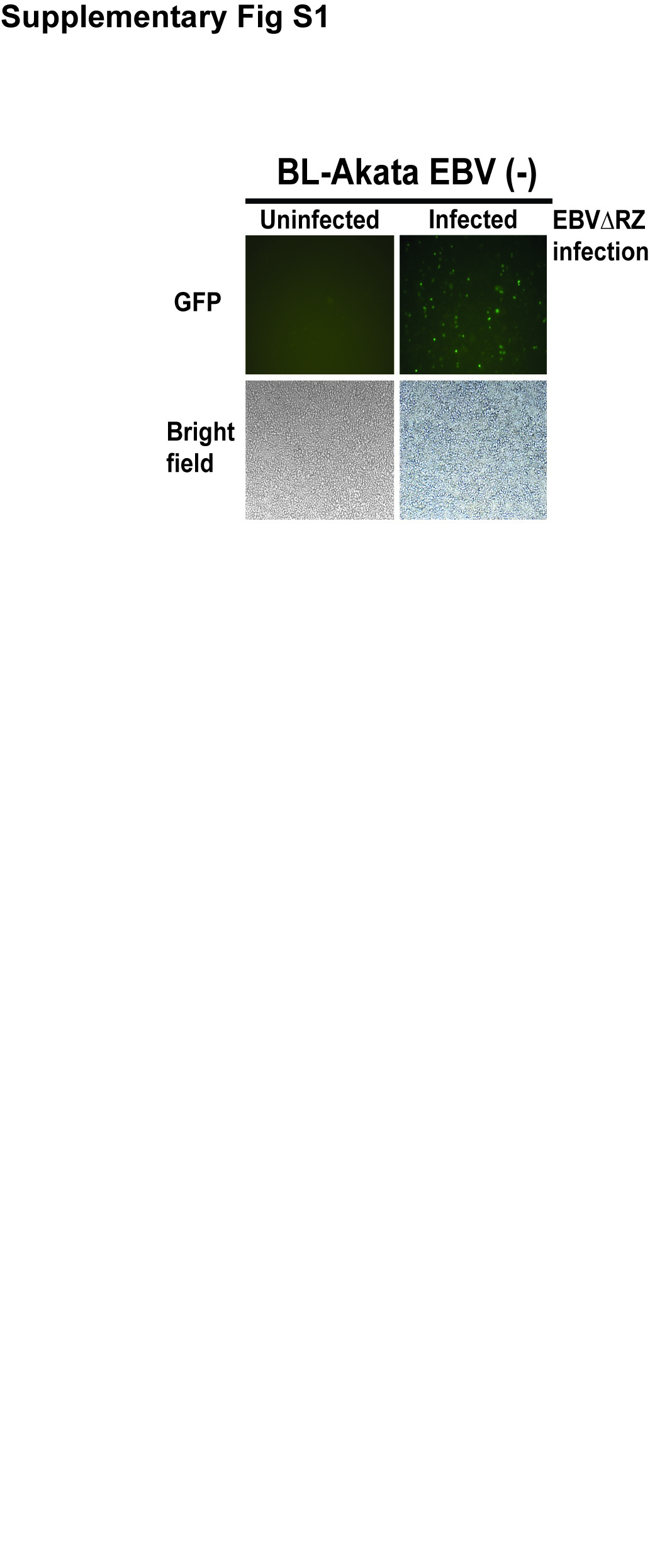

Supplement: S1 Fig — Fluorescent micrographs (top panels) and bright field images (bottom panels) of EBV-negative Akata Burkitt lymphoma (BL) cells either infected or uninfected with EBVΔRZ virions. Virions were produced from EBVΔRZ in infected HeLa cells transcomplemened with Rta and Zta. Supernatants harvested 96 hours later and passed through a 0.8 μm-pore filter to remove cellular debris. EBV-negative Akata BL cells (2X106) were then infected with this supernatant for 2 hr at 37˚C. Images were obtained forty-eight-hours post-infection. green fluorescent protein (GFP) signals derive from a reporter cassette in the EBVΔRZ virus. (TIF) [file ppat.1010886.s001.tif]

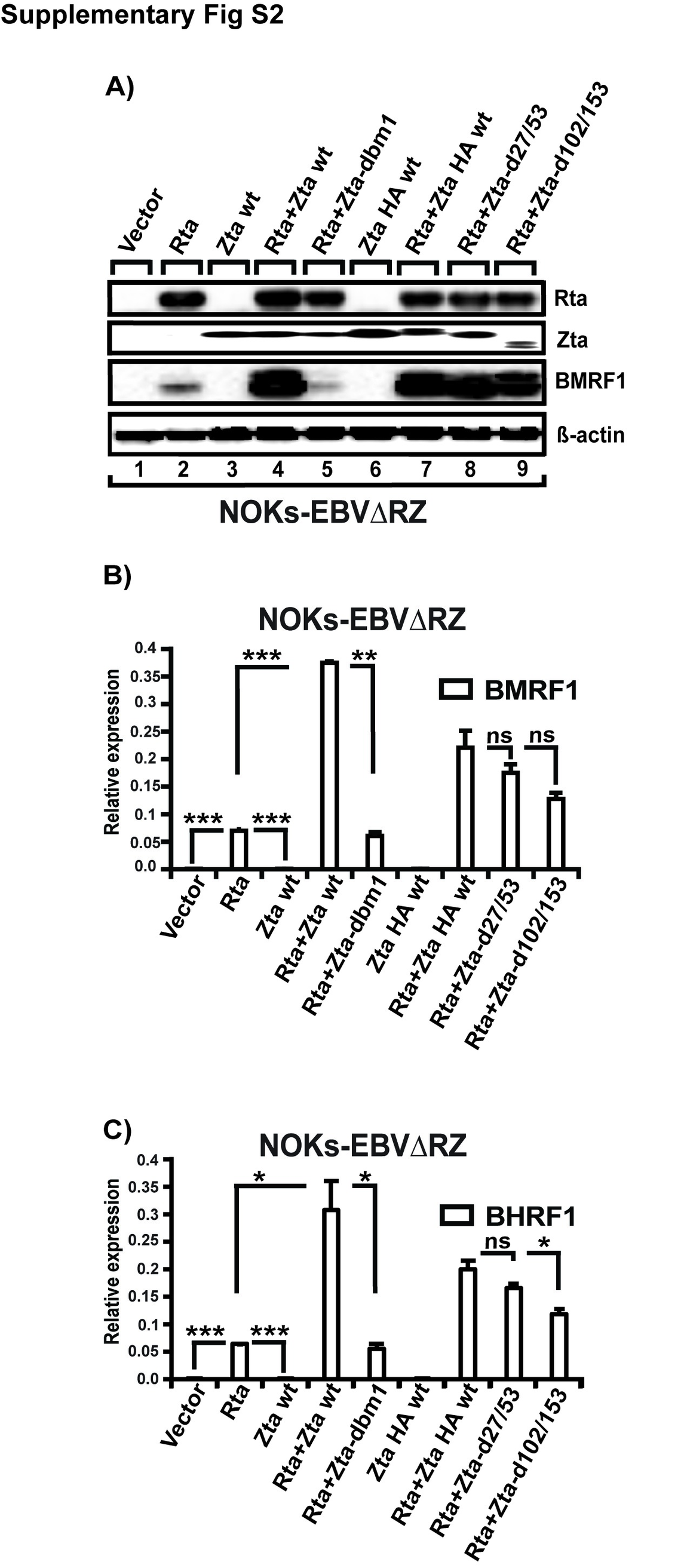

Supplement: S2 Fig — (A) Western blots for the indicated proteins from NOKs-EBVΔRZ trans-complemented with Rta alone and Rta and one of the following Zta expression constructs: wild-type Zta (Zta wt) HA-tagged Zta (Zta HA wt), and Zta K178E, R179E, Y180L (Zta-dbma1)—a ZRE-binding-defective mutant. Mutant Zta-d27/53 and Zta-d102/153 are two Zta activation domain mutants. RT-qPCR data displaying the expression of BMRF1 (B) and BHRF1 (C) in NOKs-EBVΔRZ trans-complemented as described for (A). Significant differences are indicated as follows: P ≤ 0.05 (*), P ≤ 0.01 (**), P ≤ 0.001 (***), P>0.05 (ns). (TIF) [file ppat.1010886.s002.tif]

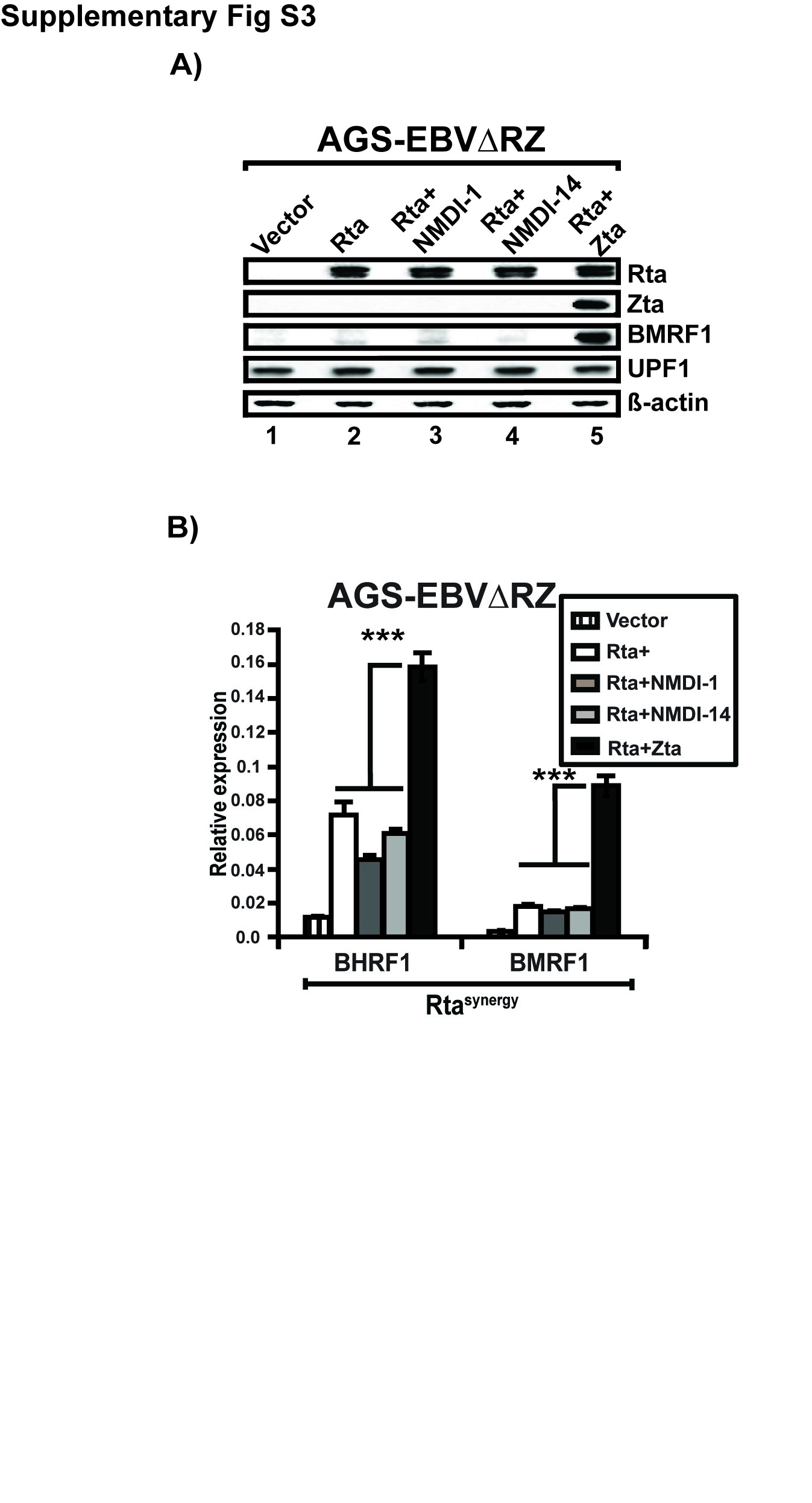

Supplement: S3 Fig — (A) Western blot of AGS-EBVΔRZ cells transfected with Rta or Rta plus Zta, and treated with the nonsense mediated decay inhibitors (NMDI-1 or NMDI-14) where indicated, then probed for the indicated EBV proteins, UPF1, and beta actin control. (B) Real-time qPCR data of the same cells described in (A) quantifying expression of two representative Rtasynergy transcripts (BHRF1 and BMRF1). Significant differences are indicated as follows: P ≤ 0.05 (*), P ≤ 0.01 (**), P ≤ 0.001 (***), P>0.05 (ns). (TIF) [file ppat.1010886.s003.tif]

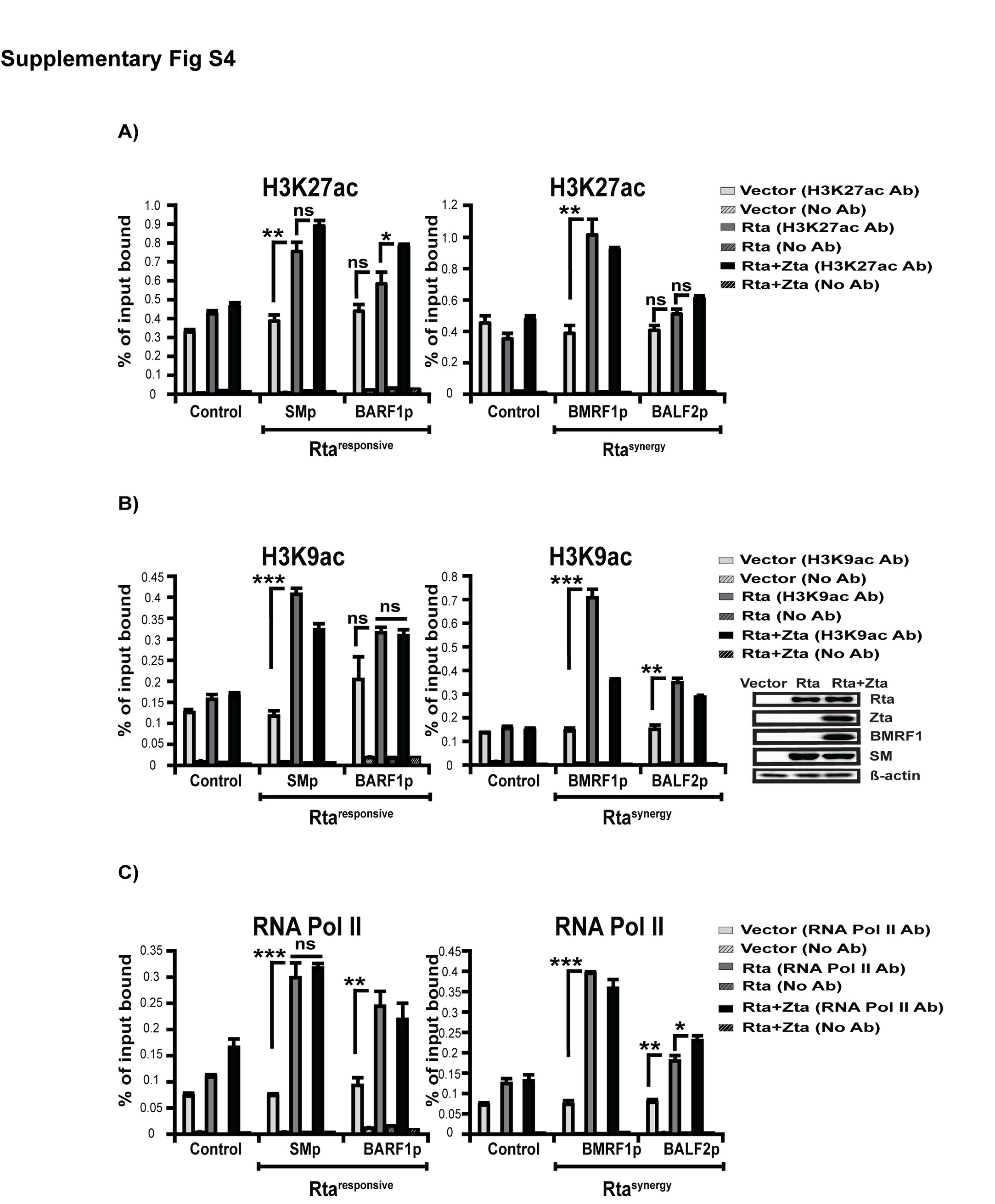

Supplement: S4 Fig — Chromatin immunoprecipitation assay for histone H3K27ac (A), H3K9ac (B), and RNA polymerase II (C) at the indicated EBV early promoters and a region of the EBV genome devoid of Rta binding (control) measured by quantitative PCR (ChIP-qPCR) in AGS-EBVΔRZ cells trans-complemented with Rta alone (dark gray bars) or with Rta and Zta (black bars). The qPCR data is reported as a percentage of the input sample with error bars indicating standard error of the mean. Chromatin samples used in (A) are from the same transfected cells shown in Fig 8B. Western blotting (middle right panel) was performed to ensure early gene induction (BMRF1 and SM) and equivalent expression of Rta in the Rta versus Rta+Zta conditions before preparing chromatin samples for the experiments done in (B) and (C). Significant differences are indicated as follows: P ≤ 0.05 (*), P ≤ 0.01 (**), P ≤ 0.001 (***), P>0.05 (ns). (TIF) [file ppat.1010886.s004.tif]

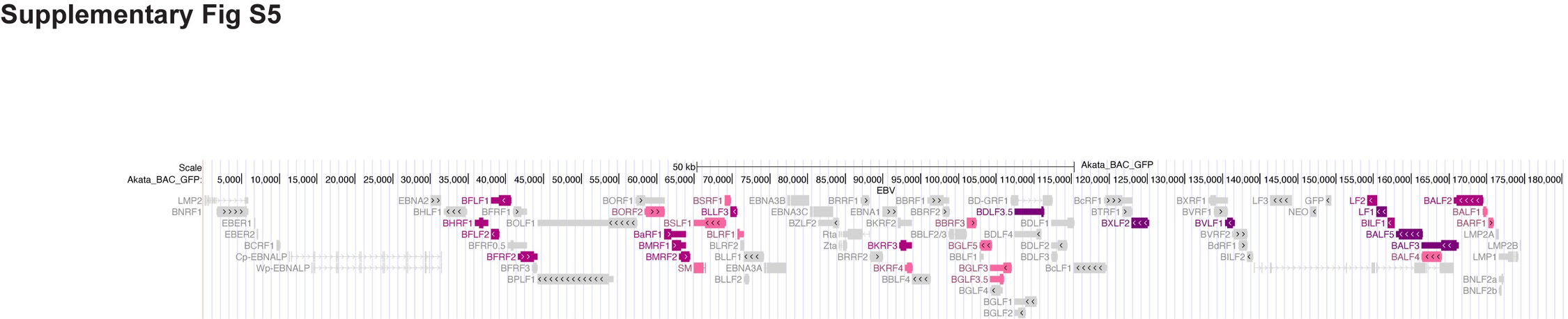

Supplement: S5 Fig — UCSC genome browser image highlighting the locations of early and leaky lytic transcript locations color coded by their Rta and Zta responsiveness. Rtaresponsive transcripts are shown in pink, Rtasynergy transcripts in light purple, and Rta+Zta in dark purple. (TIF) [file ppat.1010886.s005.tif]
